# Temperature and phosphorus: the main environmental factors affecting the seasonal variation of soil bacterial diversity in Nansi Lake Wetland

**DOI:** 10.3389/fmicb.2023.1169444

**Published:** 2023-06-30

**Authors:** Lei Chen, Yuying Shi, Shen Wang, Mengyao Sun, Meng Wang, Xiaoyue Ren, Zenghao Gao, Yiping Zhou, Jie Zhang, Weijing Zhuang, Xinyue Su, Yongchao Fu, Mengmeng Wu

**Affiliations:** ^1^College of Life Sciences, Qufu Normal University, Qufu, Shandong, China; ^2^Lunan Geo-Engineering Exploration Institute of Shandong Province, Yanzhou, Shandong, China; ^3^Shandong Freshwater Fisheries Research Institute, Jinan, Shandong, China

**Keywords:** soil microbial diversity, environmental factors, Nansi Lake Wetland, seasonal variation, available phosphorus

## Abstract

**Introduction:**

The soil bacteria promote the circulation conversion of lake nutrients and play an important role in maintaining the balance of the lake ecosystem. Few studies have investigated the association of seasonal variation in bacteria and environmental factors in inland freshwater lake wetlands. Nansi Lake is a large shallow freshwater lake in northern China. It is an important hub of the eastern route of the South-to-North Water Diversion Project.

**Methods:**

In this study, bacterial 16S *rRNA* genes were used to analyze the variation of soil bacterial community diversity in Nansi Lake Wetland and its influencing factors in different seasons.

**Results:**

It is showed that the phylum, family, and genus with the largest relative abundance in the soil of Nansi Lake Wetland are *Proteobacteria, Nitrosomonadaceae*, and *MND1*, respectively. There were significant seasonal differences in soil bacterial diversity in Nansi Lake Wetland, which was significantly higher in summer than in winter. Seasonal variation in environmental factors was significantly correlated with the variation in bacterial communities. Temperature and the content of available phosphorus may be the key factors influencing seasonal variation in bacterial diversity.

**Discussion:**

The results of this study further enhance our understanding of the relationship between bacterial community diversity and environmental factors in the lake wetland ecosystem, which can provide scientific data for the conservation of Nansi Lake Wetland.

## Introduction

As an ecotone between land and water, a wetland is the reaction pool for many important ecosystem processes. It not only does the basic functions such as supply, regulation, and support, but also is an important base for biological purification, material transformation, and environmental recovery (Mellado and Vera, 2021). Located in the south of Shandong Province, China, Nansi Lake is an important hub of the eastern route of the South-to-North Water Diversion Project. It is a typical inland shallow freshwater lake, which plays an important role in regulating water storage and adjusting the local climate. In recent years, due to the influence of human activities and the unscientific development and utilization of the lake, both the water storage of the Nansi Lake and the acreage of the natural wetland have declined (Zhang et al., 2007; Guo et al., 2019). After the running of the eastern route of the South-to-North Water Diversion Project, the water quality of the Nansi Lake has been significantly improved. However, the water in some areas of Nansi Lake still shows a certain degree of eutrophication (Zheng et al., 2023).

Lake microorganisms promote nutrient circulation and exchange through their physiological and biochemical activities, so as to promote the normal function of lake ecosystem (Quiza et al., 2014; Blanchette and Lund, 2021). Previous studies have shown that wetland soil bacterial diversity was closely related to various environmental factors and nutrient elements, such as temperature, pH, total organic carbon (TOC), total nitrogen (TN), available phosphorus (AP) and carbon/nitrogen ratio (Lin et al., 2012; Narrowe et al., 2017; Sui et al., 2019, 2021). It is found that total phosphorus and organics were the main factors affecting the bacterial community in lake wetland and estuarine sediments of Taihu Lake (Huang et al., 2019). Temperature and nitrite concentration were the main environmental factors affecting the seasonal variation of bacterial communities in the tidal flat of the Yellow River Delta (Lv et al., 2016). Sui et al. (2019) found that soil pH, AP content, nitrogen, and TOC had significant effects on soil biodiversity when they studied soil microbiota in the Sanjiang Plain wetland in northeast China. The study of microbiota in water and sediment species in Hulun Lake found that total arsenic, pH, and sulfate were the main environmental factors affecting the bacterial ecology of the water and sediments in Hulun Lake. The rapid changes in temperature, pH, and dissolved oxygen were potentially the major factors influencing seasonal bacterial diversity trends of the bacterial communities in Hulun Lake (Shang et al., 2020). Thus, correlating the changes in soil bacterial diversity with the changes in soil nutrient elements and environmental factors and looking for the environmental indicators that are closely related to the change in soil bacterial diversity are helpful to understand the role of soil microbes in the circulation and transformation process of nutrient elements in Nansi Lake Wetland, and to explore the restoration mechanism of wetland soil ecosystem function, which has theoretical significance for the protection and restoration of Nansi Lake Wetland ecosystem.

Several studies have reported about the spatial and temporal changes of soil nutrient elements in Nansi Lake Wetlands. Shu et al. (2012) studied the spatial and temporal distribution of nitrogen and phosphorus in the water of Nansi Lake and discussed the significant seasonal differences in the contents of TP in Nansi Lake. Wang et al. (2017) explored the correlation among different morphological components of phosphorus in sediments of Nansi Lake and found that organic matter in sediments of Nansi Lake was significantly correlated with TN and TP, suggesting that nitrogen and phosphorus had certain similarities in deposition behavior. However, fewer studies have concerned the soil microbes in Nansi Lake Wetland and fewer studies have been reported on the association of soil nutrient elements and soil microbes in Nansi Lake Wetland.

In this study, we collected soil samples from different seasons of the Nansi Lake Wetland to study the temporal changes of soil nutrient elements, soil environmental factors, and soil bacterial diversity. By examining the environmental factors associated with soil bacterial changes, we will explore the relationship between soil environmental factors and wetland bacterial diversity. These results will lay the foundation for evaluating the role of soil bacteria in the cycle of soil nutrient elements and for the protection of the Nansi Lake Wetland ecosystem.

## Materials and methods

### Sample collection

Four sample plots in Nansi Lake Wetland, including Xinxue plot (34°44′33″N, 117°44′23″E, plot X), Si plot (35°14′53″N, 116°40′35″E, plot S), Dongyu plot (35°0′8″N, 116°44′21″E, plot D) and Taibai plot (35°19′52″N, 116°44′21″E, plot T) (Supplementary Figure S1), were selected, and the surface soil samples at each sample plots were collected in summer (Group S) and winter (Group W) to detect soil environmental factors, content of nutrient elements, and soil bacterial diversity (Supplementary Table S1). Three repeated samples were collected in each plot and each season in 2021. Summer soil samples were collected on June 19th, 26th, and July 3rd, and winter soil samples were collected on January 7th, 14th, and 21st. After the samples were collected, the three repeated samples from each plot in the same season were mixed for further testing. We selected three flat, wet, close to the water but not flooded sample sites at each plot. The sample sites presented an equilateral triangular distribution with a spacing of about 10 m. After removing about 5-cm-thick surface plants, roots, deciduous residues, and rhizosphere soil, a 10-ml sterile sampling tube was used to collect samples for soil bacterial test. Samples were sealed and transported to the laboratory on dry ice and were then frozen at −80°C. A sterilized soil sampler was used to collect 10 kg of topsoil sample with a depth of 5–15 cm at the same sampling place to soil bacterial samples. The samples were sealed and stored in sterilized containers and were transported to the laboratory at 4°C. Soil samples were preprocessed according to the requirements of various indicators for subsequent analysis.

### Detection of soil environmental factors and soil nutrient elements and comparison among groups

Soil temperature was detected during sampling. Soil pH was detected by a standard pH meter with potentiometric determination (NY/T 1377-2007). Moisture content was detected by weight difference (NY/T 52-1987). Total phosphorus and the available phosphorus were detected by ultraviolet spectrophotometer (Beijing Purkinje General Instrument Co., Ltd). Total phosphorus was detected by alkali fusion molybdenum-antimony resistance spectrophotometry (NY/T 88-1998), and the available phosphorus was detected by sodium bicarbonate extraction-molybdenum-antimony spectrophotometry (HJ 704-2014). The total nitrogen and total organic carbon were detected by the TOC analyzer (multi N/C 3100). Total nitrogen was detected by Kjeldahl determination, and total organic carbon was detected by combustion oxidation and non-dispersive infrared determination (Shang et al., 2020; Wang et al., 2020; Sui et al., 2021). The *t*-test was used to examine significant differences in environmental factors between summer and winter groups.

### Soil genome extraction, 16S *rRNA* gene amplification, library construction, and sequencing

Genomic DNA was extracted from soil samples by CTAB method. Primers 515F/806R were used to amplify the V4 region of the 16S *rRNA* gene, with barcode attached. PCRs were performed using Phusion^®^ High-Fidelity PCR Master Mix (New England Biolabs, 15 μL). The forward and reverse primers were 2 μM each, and the template DNA was 10 ng. The thermal cycles were as follows: initial denaturation at 98°C for 1 min, denaturation at 98°C for 10 s, annealing at 50°C for 30 s, extension at 72°C for 30 s, and finally incubate at 72°C for 5 min. PCR products were detected by 2% agarose gel electrophoresis and mixed with equal density. The mixed PCR products were purified by a gel extraction kit (Qiagen). Sequencing libraries were generated using the TrUSEQ^®^ DNA PCR-Free Sample Preparation Kit (Illumina, USA) as recommended by the manufacturer, and index codes were added. The quality of the library was assessed using the Qubit 3.0 fluorometer (Thermo Scientific) and the Agilent Bioanalyzer 2100 system. Finally, the library was sequenced on an Illumina NovaSeq platform and 250-bp paired-end reads were generated.

### Alpha diversity and beta diversity analysis of soil microbiota

Paired-end reads from the original DNA fragments were merged using FLASH and assigned to each sample according to the unique barcodes. Raw data were filtered by QIIME quality filters, and the clean data were analyzed using QIIME software package. First, the clean data were used to pick operational taxonomic units (OTUs). Sequences with ≥97% similarity were assigned to the same OTU. The Venn graph was drawn according to the OTU annotation results to compare the OTU distribution among groups. Then, representative sequences for each OTU were picked and the RDP classifier was used to annotate taxonomic information for each representative sequence. The top 10 groups of abundance in each sample or group were selected to generate columnar graphs of relative species abundance. Based on the species annotation and abundance information of all samples (groups) at the genus level, the top 35 genera were selected and clustered at the species level based on the abundance information in each sample (the group abundance is the average abundance of all samples in the group), and the R software pheatmap package was used to find which species cluster more or less content in which samples.

QIIME software was used to calculate alpha diversity indices, including observed species, Chao1, Shannon, Simpson, ACE, and Good's coverage. Wilcoxon rank-sum test was performed using R software to evaluate the differences among the alpha diversity indices of different groups. Beta diversity analysis was also performed on QIIME software on both weighted and unweighted unifrac distance to build the cluster trees. The rationality of the grouping was detected by Anosim and ADONIS tests. The comparison of the abundance differences of annotated species between groups under different taxonomic levels was calculated by MetaStat tests. Cluster analysis was preceded by principal component analysis (PCA), principal coordinate analysis (PCoA), and non-metric multidimensional scaling (NMDS) and displayed by ggplot2 package in R software. Linear discriminant analysis effect size (LEfSe) software was used to detect the difference in the abundance of each rank of classification (phylum, class, order, family, and genus) between groups and to find the biomarkers with key contributions to the difference between groups.

### Functional annotation of soil bacterial genes and comparison between groups

Functional annotation based on the KEGG database was performed using the PICRUSt software package. The Venn Graph was drawn to compare the gene function distribution among groups. The functional information with the top 10 maximum abundance at each annotation level for each sample or group was selected to generate a column graph of functional relative abundance. The top 35 abundant functions and their relative abundance information in each sample were selected to draw the heatmaps and then clustered from the level of functional differences. Dimensionality reduction analysis (PCoA, PCA, NMDS analyses) and cluster analysis (UPGMA clustering) were performed based on the functional annotation results to detect the similarity of the bacterial functional composition in each group.

### Analysis of the relationships between environmental factors and bacterial communities

Spearman's correlation coefficient was used to explore the relationships between bacterial communities and environmental factors. Redundancy analysis (RDA) and the “envfit” function with 999 permutations in R vegan package were used to reveal the significant correlations between the bacterial communities and environmental factors (Dawson et al., 2012).

## Results

### Soil environment factors of Nansi Lake Wetland

The soil in the Nansi Lake Wetland is weakly alkaline. The pH values of different sample plots are from 8.07 to 8.62. There is no significant difference in the pH value between winter and summer groups. The temperature and TP content in summer were significantly higher than those in winter (*P* < 0.05, *F* = 749.568, *F* = 4.549), while the TOC content in winter was significantly higher than that in summer (*P* < 0.05; *F* = 3.013), and AP content in winter was extremely significantly higher than that in summer (*P* < 0.01; *F* = 30.109) (Supplementary Table S2).

### Soil bacterial composition in Nansi Lake Wetland

After filtering and removing chimeras, a total of 1,455,990 effective tags were obtained from the soil samples of Nansi Lake Wetland. The rarefaction curve and species accumulation boxplot tended to be flat, indicating that the sequencing data can reflect the microbial diversity in soil samples (Supplementary Figure S2). A total of 14,981 OTUs were obtained by clustering with 97% similarity. After species annotation, 116 phyla (including 12 archaea and 104 bacteria), 235 bacterial classes, 453 bacterial orders, 619 bacterial families, and 989 bacterial genera were identified in total.

The phyla with the highest relative abundance in soil bacteria of Nansi Lake Wetland are *Proteobacteria* (30.87%), followed by *Acidobacteriota* (8.30%), *Actinobacteriota* (6.65%), *Firmicutes* (5.65%), *Chloroflexi* (3.71%), *Cyanobacteria* (3.69%), *Bacteroidota* (2.46%), *Desulfobacterota* (2.20%), *Methylomirabilota* (1.73%), etc. The most abundant family in soil bacteria of Nansi Lake Wetland is *Nitrosomonadaceae* (6.00%), followed by Gemmatimonadaceae (4.11%), *Rhizobiaceae* (3.78%), Comamonadaceae (2.89%), Vicinamibacteraceae (2.32%), etc. The most abundant genus is *MND 1* (4.31%), followed by *Thiobacillus* (1.90%), *Streptococcus* (1.10%), *Lactobacillus* (1.07%), *Pseudomonas* (1.01%), etc.

**Figure 1:**
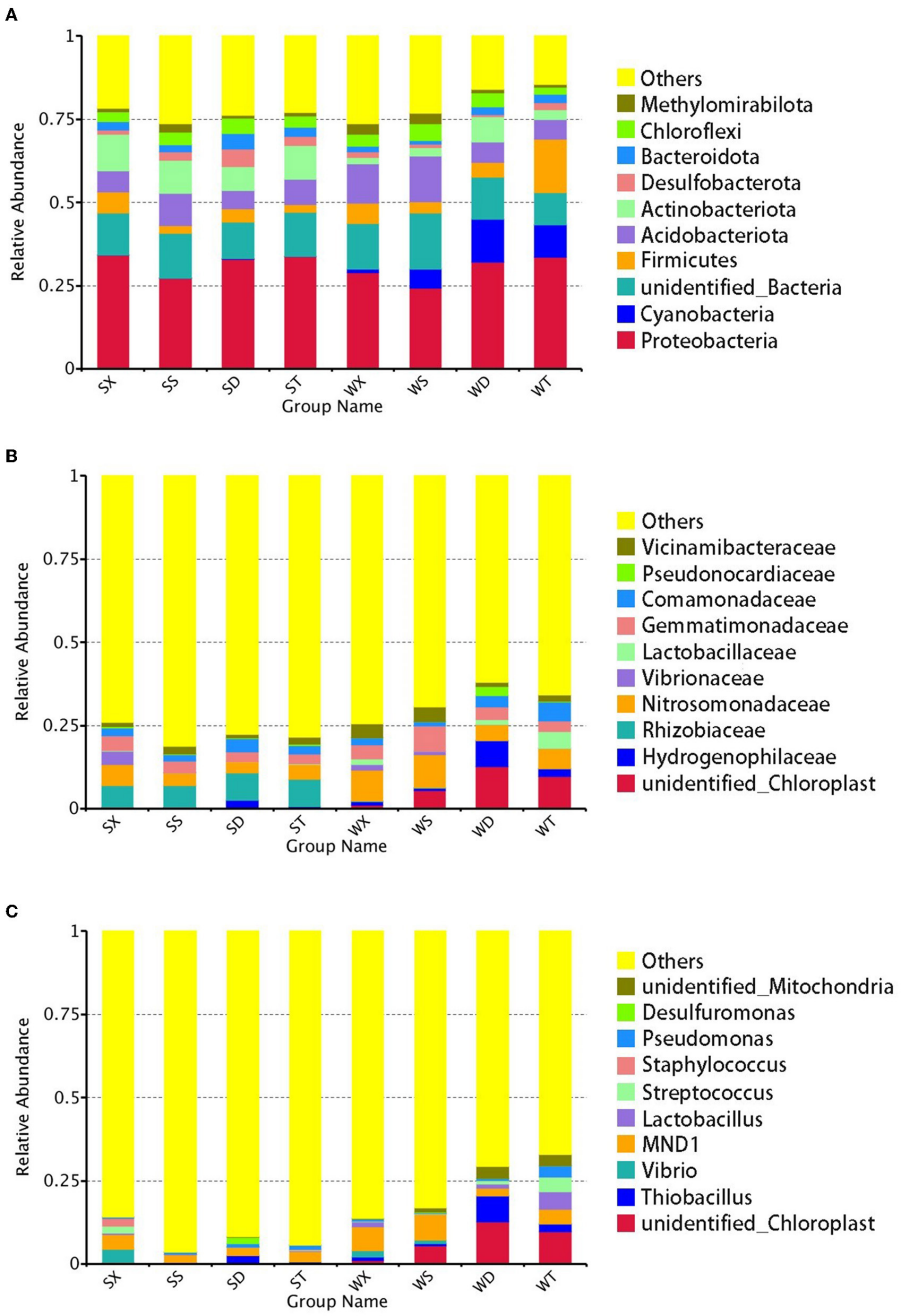
Relative abundance of annotated genes at phylum, family, and genus levels. The top 10 phylum **(A)**, family **(B)**, and genus **(C)** with the highest abundance in each group were selected, and the rest were set to others.

In summer, 8.41% of total OTUs were unclassified at the phylum level, while at the family and genus levels, they are 32.58 and 60.95%, respectively. The most abundant phylum in summer is *Proteobacteria* (32.03%), and the most abundant family and genus are *Rhizobiaceae* (7.44%) and *MND1* (3.20%), respectively. In winter, 6.92% of total OTUs were unclassified at the phylum level, while at the family and genus levels, they are 29.14 and 51.65%, respectively. The phylum with the highest relative abundance in winter is also *Proteobacteria* (29.71%). But the most abundant family in winter is *Nitrosomonadaceae* (7.51%), and *unidentified_Chloroplast* is the most abundant genus in winter (7.26%), while the genus *MND1* still has a high relative abundance (5.43%) (Figure 1).

### Soil bacterial alpha and beta diversity analysis

Alpha diversity analysis found that the observed species, Chao1, ACE, and Shannon indices of Group S were significantly higher than those of Group W (*P* < 0.01), which indicated that soil bacterial diversity of Nansi Lake Wetland was higher in summer than that in winter (Supplementary Table S3).

**Figure 2:**
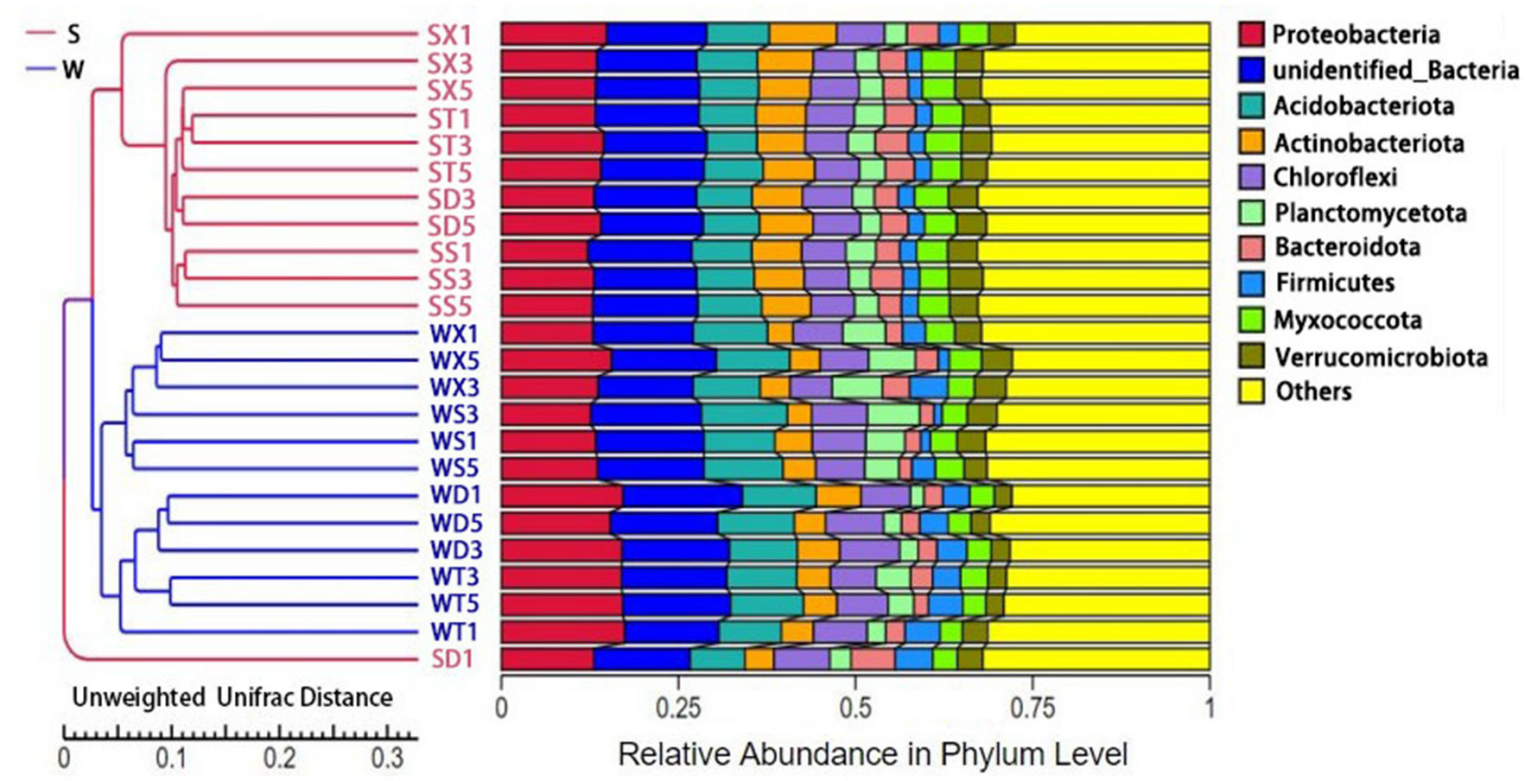
UPGMA cluster analysis results based on species abundance.

The results of PCoA and PCA based on annotated species abundance of soil bacteria showed that samples from summer and winter groups were aggregated separately. NMDS (non-metric multi-dimensional scaling) analysis based on Bray–Curtis distance showed that there were significant differences in bacterial community diversity between Group S and Group W (*P* = 0.001) (Supplementary Figure S3). UPGMA cluster analysis showed that samples from Group S clustered together, while samples from Group W clustered into another clade (Figure 2). There was no obvious aggregation of samples from the same sampling point in different seasons, but samples from different points in the same season gathered together, indicating that the seasonal difference in soil bacterial diversity was more significant. The results of Anosim and Adonis analysis based on Bray–Curtis distance also showed that the differences between Group S and Group W were significantly greater than the differences within groups (*P* < 0.05) (Supplementary Figure S4).

**Figure 3:**
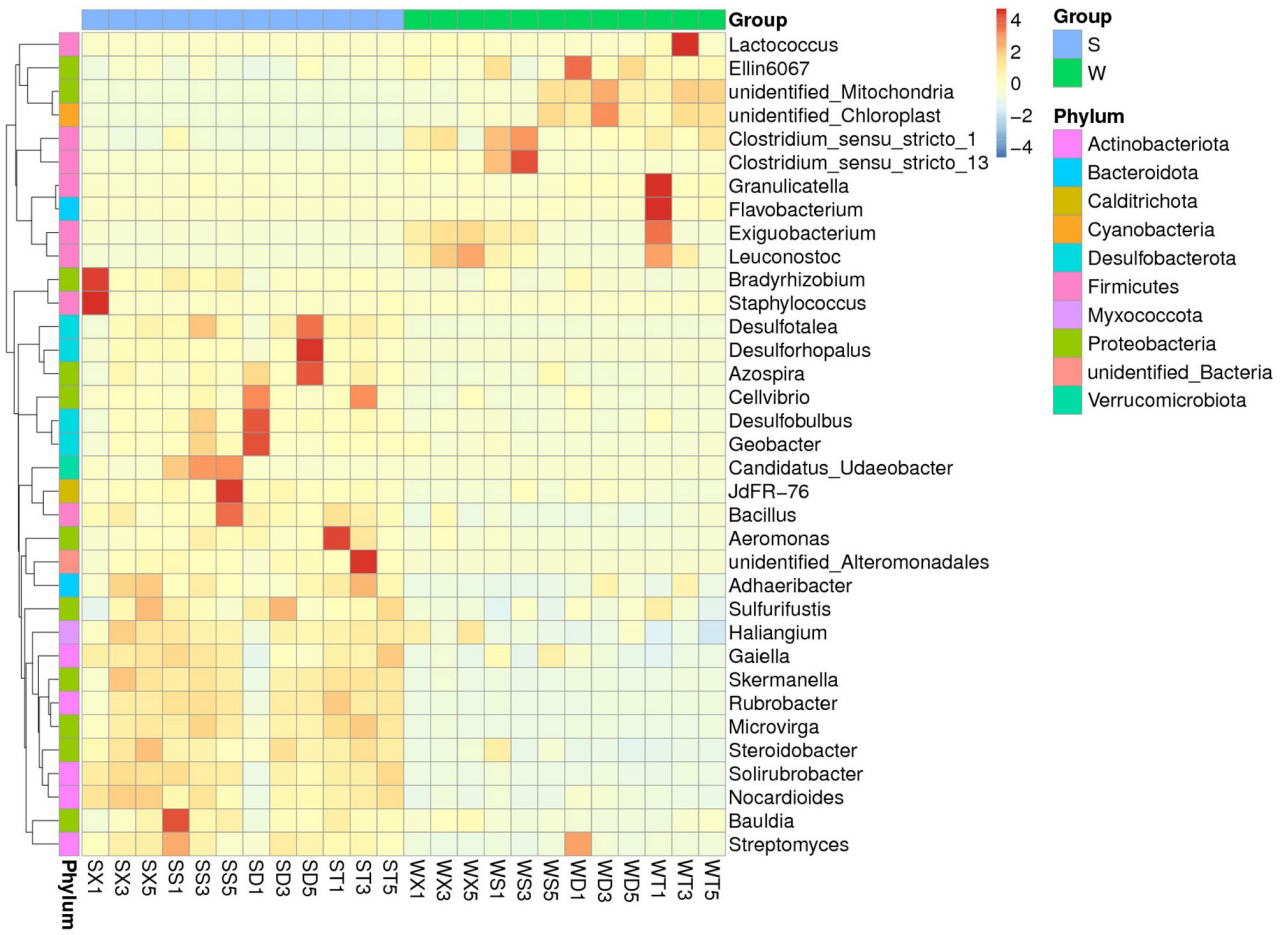
MetaStat test showed the genera with significant abundance differences between summer and winter groups.

MetaStat tests for differences between groups at each taxonomic level found that the abundances of phyla *Cyanobacteria* and GAL15 were significantly higher in winter than in summer (*P* < 0.05), while the abundances of phyla *Actinobacteriota, Desulfobacterota, Myxococcota, Nitrospirota, Calditrichota*, etc., were significantly higher in summer than in winter (*P* < 0.05). At the family level, there are significant differences between the summer and winter groups in the abundance of families *Nitrosomonadaceae, Mitochondria, Steroidobacteraceae, Clostridiaceae, Enterobacteriaceae* (Group S < Group W), and *Rhizobiaceae, Staphylococcaceae, Micrococcaceae, TRA3-20, Solirubrobacteraceae* (Group S > Group W), etc. At the genus level, the genera with significant difference between summer and winter groups are *Flavobacterium, Lactococcus, Ellin6067, Exiguobacterium* (Group S < Group W), and *Staphylococcus, Rubrobacter, Bacillus, Solirubrobacter* (Group S > Group W), etc. (Figure 3; Supplementary Figures S5, S6).

**Figure 4:**
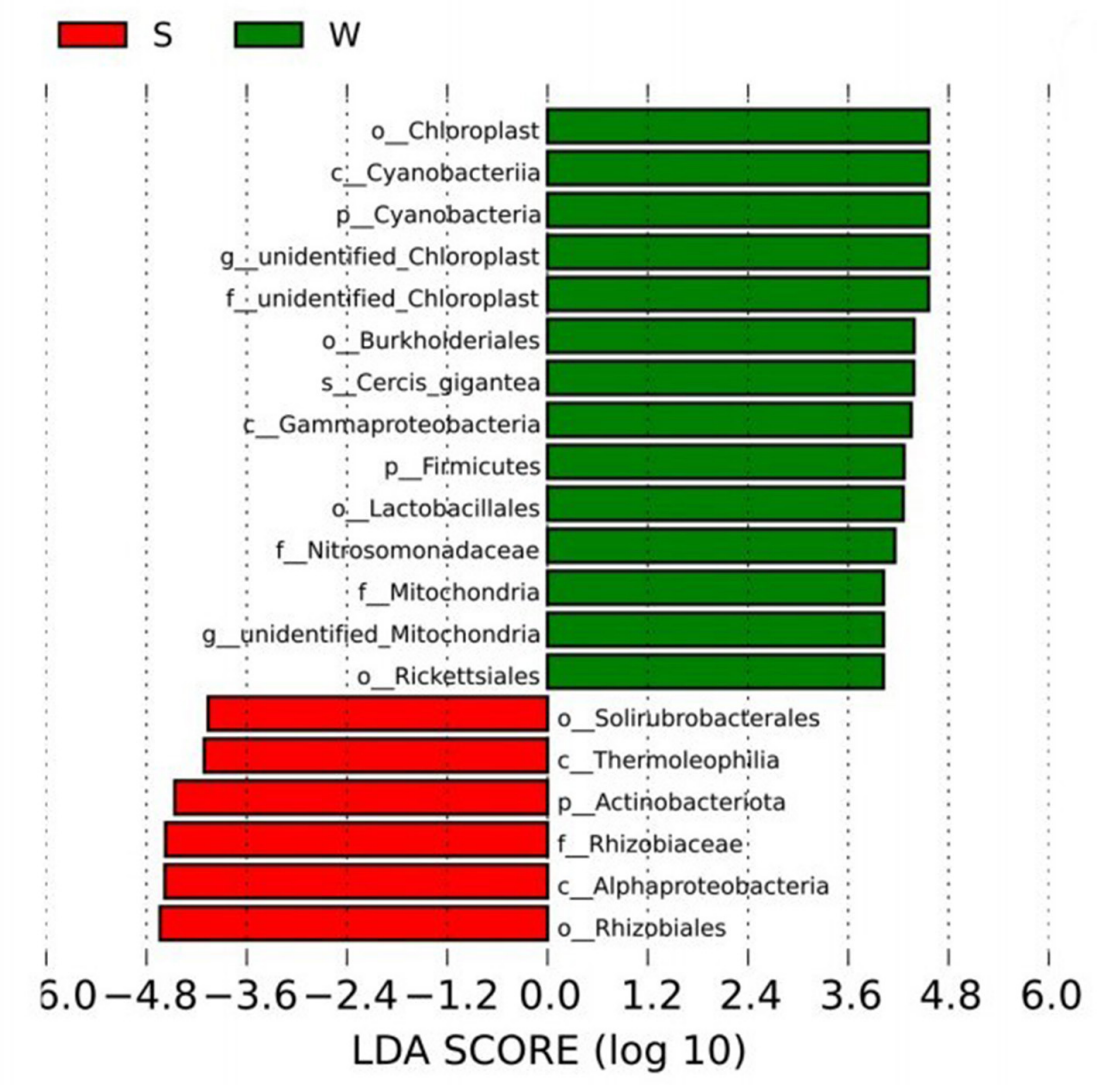
LEfSe analysis showed the biomarkers with significant differences between summer and winter groups.

The biomarkers with significant differences between groups were further searched by LEfSe analysis. The biomarkers in Group S include phylum *Proteobacteria* class Alpha*Proteobacteria* order *Rhizobiales* family *Rhizobiaceae* and phylum *Actinobacteriota* class *Thermoleophilia* order *Solirubrobacterales*. The Biomarkers in Group W include phylum *Firmicutes* class *Bacilli* order *Lactobacillales*, phylum *Proteobacteria* class *Gammaproteobacteria* order *Burkholderiales* family *Nitrosomonadaceae*, phylum *Proteobacteria* class *Alphaproteobacteria* order *Rickettsiales* family Mitochondria and *Cercis gigantea*, which belongs to the phylum *Cyanobacteria* class *Cyanobacteria* order *Chloroplast*, etc. (Figure 4).

By SIMPER analysis, it is concluded that the bacteria with high contribution to the difference between the summer and winter groups included the phyla *Cyanobacteria* (with a relative abundance of 3.61%), *Actinobacteriota* (3.41%), *Proteobacteria* (3.26%), *Firmicutes* (3.00%), *Acidobacteriota* (1.96%), *Desulfobacterota* (0.94%), *Bacteroidota* (0.87%), *Chloroflexi* (0.72%), *Methylomirabilota* (0.69%), *Myxococcota* (0.65%), etc. At the family level, the families with high contribution include *Rhizobiaceae* (3.66%), *Nitrosomonadaceae* (1.87%), *Hydrogenophilaceae* (1.35%), *Streptococcaceae* (1.13%), *Mitochondria* (1.06%), *Comamonadaceae* (1.05%), *Lactobacillaceae* (1.01%), *Gemmatimonadaceae* (0.91%), *Vicinamibacteraceae* (0.87%), *Vibrionaceae* (0.82%). The genus with high contribution include *MND1* (1.64%), *Thiobacillus* (1.35%), *Lactobacillus* (1.01%), *Streptococcus* (0.93%), *Vibrio* (0.81%), *Pseudomonas* (0.50%), *RB41* (0.47%), *Rubrobacter* (0.39%), *Sphingomonas* (0.34%), *Ellin6067* (0.32%), etc. (Supplementary Figure S7).

### Prediction and comparison of soil bacterial function

**Figure 5:**
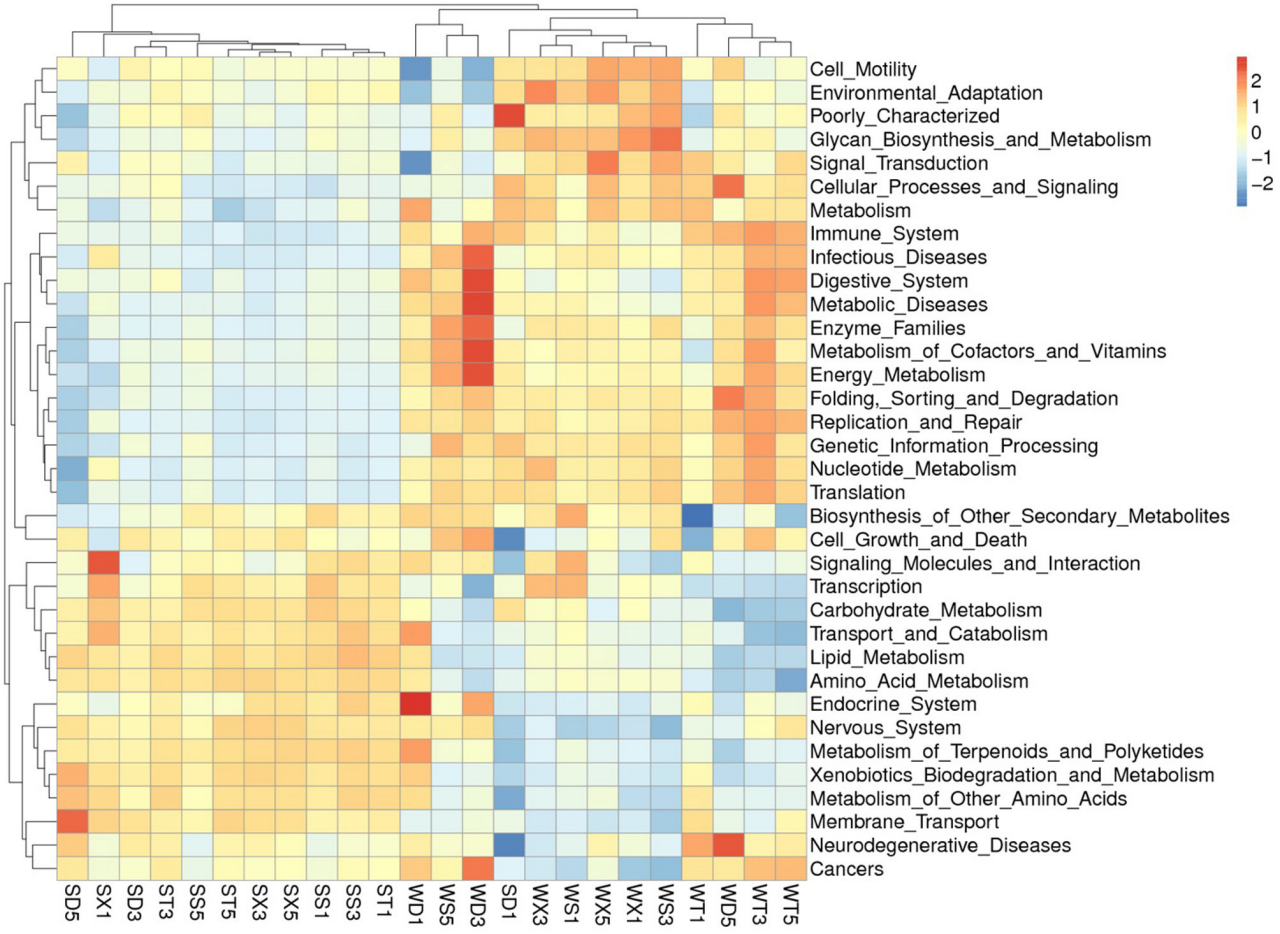
Heat map shows the annotated functions at KEGG level 2.

The KEGG database annotated functional abundance of each group shows that the function with the highest abundance is metabolism (50.08% in Group S; 49.44% in Group W), including amino acid metabolism (10.54% in Group S; 10.09% in Group W), carbohydrate metabolism (10.06% in Group S; 9.61% in Group W), energy metabolism (5.95% in Group S; 6.52% in Group W), cofactor and vitamin metabolism (4.23% in Group S; 4.42% in Group W), and lipid metabolism (3.82% in Group S; 3.54% in Group W). The second most abundant function is genetic information processing (15.85% in Group S; 16.97% in Group W), including replication and repair (6.82% in Group S; 7.38% in Group W), etc. The third is Environmental information processing (14.24% in Group S; 13.20% in Group W), including membrane transport (11.77% in Group S; 10.66% in Group W), signal transduction (3.54% in Group S; 3.71% in Group W), and cell conduction (4.37% in Group S; 4.43% in Group W). Other functions account for no more than 1%. In addition, 13.57% (Group S) to 13.99% (Group W) of unclassified bacterial functions were annotated (Figure 5; Supplementary Figure S8).

PCA based on KEGG database annotation results showed that the samples from Group S and Group W were gathered separately. The *t*-test results showed that the annotated functions with significant differences at level 1 include Metabolism, Environmental Information Processing (Group S > Group W); and Genetic Information Processing, Cellular Processes, Human Diseases, Organismal Systems (Group S < Group W), etc. The functions with significant differences at level 2 mainly included membrane transport, amino acid metabolism, carbohydrate metabolism), carbohydrate metabolism, lipid metabolism (Group S > Group W); and energy metabolism, metabolism of cofactors and vitamins, metabolism of cofactors and vitamins, nucleotide metabolism, replication, and repair (Group S < Group W), etc. (Supplementary Figures S9–S12).

### Correlation analysis between soil environmental factors and bacterial diversity

**Figure 6:**
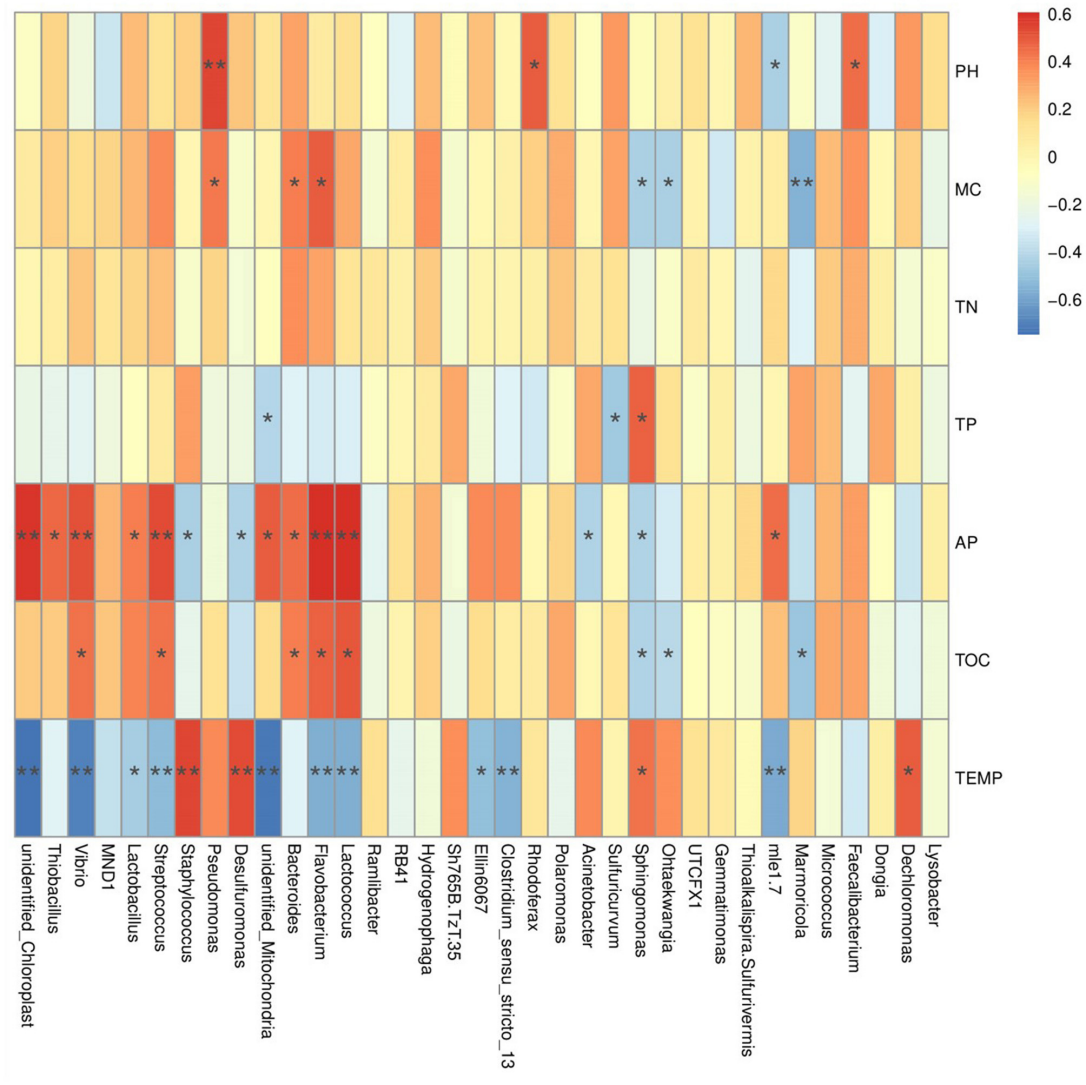
The result of Pearson correlation analysis between the abundance of annotated families and the environmental factors.

Pearson correlation analysis between soil environmental factors and bacterial communities shows that the abundances of phyla *Nitrospinota, Bdellovibrionota, Spirochaetota, Entotheonellaeota, Calditrichota, Nitrospirota, Myxococcota, Desulfobacterota*, etc., are significantly positively correlated with the temperature (*P* < 0.01). Among them, the abundances of phylum Calditrichota are extremely significantly negatively correlated with the AP content (*P* < 0.01), and the abundances of *Desulfobacterota, Myxococcota*, and *Spirochaetota* are significantly negatively correlated with the AP content (*P* < 0.05). In contrast, the abundance of phylum *Cyanobacteria* is significantly negatively correlated with temperature (*P* < 0.01) but extremely significantly positively correlated with the AP content (*P* < 0.01). The abundance of *Actinobacteriota* was significantly positively correlated to temperature and the TP content (*P* < 0.05), but significant negative correlation with the AP content (*P* < 0.05). The abundance of *Planctomycetes* was significantly positively correlated with the TP content (*P* < 0.01), but significantly negatively correlated with the pH value (*P* < 0.05) (Supplementary Figure S13A). At the family level, the abundances of *Pseudonocardiaceae, Rhizobiaceae, Rhodocyclaceae*, and *Staphylococcaceae* are extremely significant positively correlated to the temperature (*P* < 0.01), but significantly negatively correlated to the AP content (*P* < 0.05). In contrast, the abundances of *Enterobacteriaceae, Clostridiaceae, Steroidobacteraceae, Mitochondria, Streptococcaceae, Vibrionaceae*, and *Nitrosomonadaceae* are extremely significantly negatively correlated to the temperature (*P* < 0.01), but significantly positively correlated to the AP content (*P* < 0.05). The abundances of *Muribaculaceae* and *Flavobacteriaceae* are extremely significantly positively correlated to the TOC content (*P* < 0.01). The abundance of *Pseudomonadaceae* is extremely significantly positively correlated to the pH value (*P* < 0.01) (Figure 6). At the genus level, the abundance of *Desulfuromonas* and *Staphylococcus* was extremely significantly positively correlated to the temperature (*P* < 0.01), but significantly negatively correlated to the AP content (*P* < 0.05). In contrast, the abundances of *Clostridium_sensu_stricto*_13, *Lactococcus, Flavobacterium, Streptococcus*, and *Vibrio* are extremely significantly negatively correlated to the temperature (*P* < 0.01), but significantly positively correlated to the AP content (*P* < 0.01) (Supplementary Figure S13B).

Spearman's correlation analysis between soil environmental factors and alpha diversity indices shows that observed species and PD whole tree indices are significantly positively correlated with temperature (*P* < 0.01) and significantly negatively correlated with AP content (*P* < 0.01). Chao1 and ACE indexes are significantly positively correlated with temperature (*P* < 0.01) but significantly negatively correlated with TOC (*P* < 0.05) and AP content (*P* < 0.01). Shannon index is significantly positively correlated with temperature (*P* < 0.01) but significantly positively correlated with TP content (*P* < 0.05). Good's coverage is significantly negatively correlated with temperature (*P* < 0.01), but significantly positively correlated with TOC (*P* < 0.05) and AP content (*P* < 0.01) (Supplementary Figure S14).

**Figure 7:**
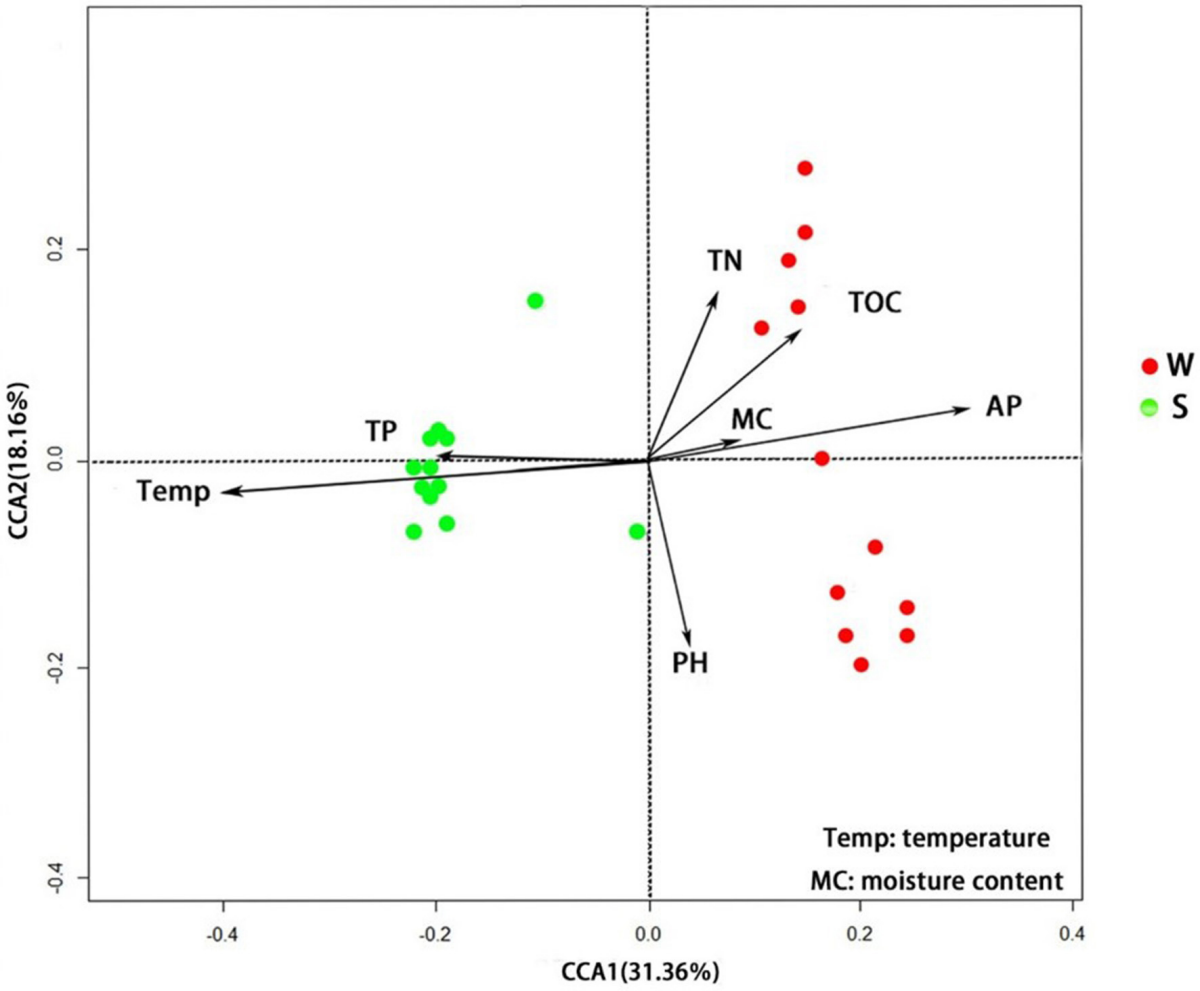
RDA shows the influence of environmental factors on soil bacterial OTU abundance.

The results of RDA show that samples collected in summer are mainly concentrated in quadrants 2 and 3, while samples from the winter group are mainly concentrated in quadrants 1 and 4. The interpretation rates of RDA axis 1 and axis 2 are 31.36 and 18.16%, respectively. Temperature (*P* < 0.01) and TP content (*P* < 0.05) are significantly correlated with soil bacterial abundance in Nansi Lake Wetland in summer, while AP content (*P* < 0.01) is significantly correlated with bacterial abundance in winter (Figure 7).

## Discussion

### Effect of environmental factors on seasonal variation of soil bacteria with higher abundance in Nansi Lake Wetland

*Proteobacteria* is a common bacterial phylum with high abundance in wetland soil (Shao et al., 2013; Wu et al., 2021). It is the most abundant phylum in the soil of Nansi Lake Wetland. Most of the bacteria in this phylum can participate in the metabolism of biodegradable organics and play an important role in biological nitrogen and phosphorus removal (Wilms et al., 2006; Nguyen et al., 2011; Shao et al., 2013; Shang et al., 2020). The Nansi Lake is at a certain level of eutrophication (Wang et al., 2017); the high abundance of *Proteobacteria* in Nansi Lake Wetland may be related to the high concentration of N and P in soil. *Acidobacteriota* also has a high prevalence and diversity in soil (Barns et al., 2007) and plays an important role in polymer degradation (Shang et al., 2020). They can inhabit natural environments with different temperature, salinity, and acidity (Pankratov and Dedysh, 2010). The degradation ability and selectivity of different species of *Acidobacteriota* to polymers such as carbohydrates are related to the types and activities of the enzymes they contain (Pankratov and Dedysh, 2010). The abundance of *Actinobacteriota* in the soil of Nansi Lake showed a significant difference between summer and winter. Spearman analysis found that the abundance of *Actinobacteriota* was significantly positively correlated with temperature and TP content and negatively correlated with AP content. Gao et al. found that the abundance of *Actinobacteriota* in the soil of the Yellow River Delta was also positively correlated with TP content (Sun et al., 2020), which is consistent with the present study. However, studies on soil bacterial diversity in northeast China found that the relative abundance of *Actinobacteriota* was significantly negatively correlated with soil temperature (Cai F. et al., 2021; Cai L. et al., 2021). Studies on the Loess Plateau found that the abundance of *Actinobacteriota* was positively correlated with the content of AP and negatively correlated with TOC and TN (Dang et al., 2017). However, in the present study, the abundance of *Actinobacteriota* had no significant correlation with the content of TOC and TN.

*Cyanobacteria* and *Desulfobacterota* are the other two phyla with high abundance in the soil of Nansi Lake Wetland. The abundance of *Cyanobacteria* in summer was significantly lower than that in winter. Spearman analysis found that the abundance of *Cyanobacteria* was significantly negatively correlated with temperature. Studies on the water of Hulun Lake also found that *Cyanobacteria* was significantly negatively correlated with temperature (Shang et al., 2022), which was consistent with the present findings. In the present study, it was found that the abundance of *Cyanobacteria* was significantly positively correlated with AP content. Phosphorus is an essential element for the genome DNA synthesis of *Cyanobacteria* and plays an important role in the growth of *Cyanobacteria* (Ren et al., 2017). In addition, *Cyanobacteria* are important nitrogen-fixing organisms (Berman-Frank et al., 2003; Prasitwuttisak et al., 2022). Both heterocytic and non-heterocytic *Cyanobacteria* can use azotase to catalyze nitrogen for biological nitrogen fixation (Bothe et al., 2010). However, no correlation was found between the abundance of *Cyanobacteria* and soil TN in the present study. The abundance of *Desulfobacterota* in Nansi Lake Wetland is extremely significantly positively correlated with temperature and significantly negatively correlated with AP content. Zhang et al. (2022) have also found that the presence of AP reduces the abundance of *Desulfobacterota*. Another study found that the decrease in TN content in soil promoted the growth of *Desulfobacterota* (Fan et al., 2022).

*Nitrosomonadaceae* is the most abundant bacterial family in the soil of Nansi Lake Wetland. *Nitrosomonadaceae* is related to the nitrogen cycle and is an important component of soil nitrite bacteria (Cua and Stein, 2011). The bacteria in *Nitrosomonadaceae* can oxidize ammonia into nitrous acid under aerobic conditions, which is the first step in the nitrification process (Avrahami and Bohannan, 2007). It is found that *Nitrosomonadaceae* tended to grow and participate in nitrification in environments with higher pH values (pH 6–9) (Kowalchuk and Stephen, 2001; Fernandez et al., 2016; Griffith et al., 2017; Pang et al., 2021). The average soil pH value of Nansi Lake Wetland is 8.29, which is conducive to the growth of *Nitrosomonadaceae*. Metastat tests found that the abundance of *Nitrosomonadaceae* in Nansi Lake Wetland soil was significantly lower in summer than in winter (*q* < 0.05). Spearman correlation analysis also showed that the abundance of *Nitrosomonadaceae* was significantly negatively correlated with temperature (*P* < 0.01). Avrahami and Bohannan (2007) found that the overall abundance of *Nitrosomonadaceae* decreased with increasing soil temperature. This is consistent with the present studies in Nansi Lake Wetland. They also found that the increase in the interaction of soil temperature, moisture content, and fertilizer could reduce the relative abundance of some bacteria of *Nitrosomonadaceae* (such as Nitrosospira sp.) (Avrahami and Bohannan, 2007). In the present study, we also found that the abundance of *Nitrosomonadaceae* was significantly positively correlated with AP content. This was proved by previous studies on soil in northeast China (Cai F. et al., 2021; Cai L. et al., 2021).

*Rhizobiaceae* is another family with high abundance in the soil of Nansihu wetland. Contrary to *Nitrosomonadaceae*, the *Rhizobiaceae* abundance in Nansi Lake Wetland was significantly higher in summer than in winter (*q* < 0.01). Correlation analysis showed that the abundance of *Rhizobiaceae* was significantly positively correlated with temperature (*P* < 0.01) and significantly negatively correlated with AP content (*P* < 0.01). Bacteria in *Rhizobiaceae* promote soil nitrogen circulation mainly by participating in nitrogen fixation in the soil (Narendrula-Kotha and Nkongolo, 2017), which is significant for increasing soil nitrogen content and promoting plant growth (Vuko et al., 2020). *Rhizobium*, a genus in *Rhizobiaceae*, contains key groups associated with the nitrogen cycle and is a major contributor to the global nitrogen cycle through symbiotic nitrogen fixation with various plants (Narendrula-Kotha and Nkongolo, 2017; Abdul Rahman et al., 2021; Gui et al., 2022). *Rhizobiaceae* can also dissolve precipitated P-metal complexes and release inorganic phosphorus to increase soil phosphorus content, thus regulating soil phosphorus cycling (Sun et al., 2020). The higher abundance of *Rhizobiaceae* in Nansi Lake Wetland is closely related to nitrogen and phosphorus cycling in the soil.

### Seasonal variation of temperature, AP, and TP had significant effects on bacterial diversity in Nansi Lake Wetland

There were significant seasonal differences in soil environmental factors in Nansi Lake Wetland. Temperature and TP content were significantly higher in summer than in winter, while AP and TOC contents were significantly higher in winter than in summer. Comparison of alpha diversity indices found that the observed species, Chao1, ACE, and Shannon indices of soil microorganisms in summer were significantly higher than those in winter (*P* < 0.01), indicating a higher diversity of soil microbiota in summer. Previous studies also found that the abundance of soil bacterial richness increased significantly with the increase in temperature (Sierra et al., 2015; Wang et al., 2015). RDA showed that the seasonal variation of environmental factors, such as temperature, AP, and TP, had significant effects on the bacterial community in Nansi Lake Wetland. Bacterial community diversity in summer was significantly correlated with temperature and TP content (*P* < 0.05), while bacterial community diversity in winter was significantly correlated with AP content (*P* < 0.01). Spearman correlation analysis found that soil bacterial alpha diversity indices (such as observed species, ACE, Chao1) of Nansi Lake were significantly positively correlated with the temperature, but significantly negatively correlated with soil AP content. These results indicated that the higher AP content in Nansi Lake soil in winter was an important reason for the lower soil bacterial diversity. As the difference in pH value between winter and summer is not significant, pH value has no significant effect on soil bacterial diversity in Nansi Lake Wetland.

## Conclusion

In this study, we compared the soil bacterial diversity of Nansi Lake Wetland in different seasons, correlated the relationship between bacterial diversity and environmental factors, and found that besides temperature, AP and TP in soil are the key factors affecting bacterial diversity. These results provide a scientific reference for the ecological protection and management of Nansi Lake Wetland. However, there are still some limits in the present study. First, the present study discussed the soil bacterial diversity and soil environmental factors in winter and summer. Long-term inter-annual surveys will facilitate systematic monitoring of dynamic changes in the soil environment and soil microbiota. Second, this study discussed the influence of the environment on microorganisms, while the function of microorganisms and their effect on the environment need to be further studied in order to better explore the relationship between community succession of soil microbiota and the development of soil environment in the Nansi Lake Wetland. Third, in this study, the function of soil bacteria and its relationship with environmental factors were achieved by functional prediction. In future, these differential taxa will be validated by isolating representative bacteria (Jin et al., 2023), so as to further evaluate the role of soil microbiota in the wetland ecosystem.

## Data availability statement

The datasets presented in this study can be found in online repositories. The names of the repository/repositories and accession number(s) can be found below: https://www.ncbi.nlm.nih.gov/, PRJNA871950.

## Author contributions

LC conceived and designed the experiments, collected samples, and modified the manuscript. LC and MWu contributed to the fund. YS and SW performed the experiments, analyzed the data, and wrote the manuscript. YS and XR plotted the figures in the manuscript. MS, MWa, ZG, YZ, JZ, WZ, XS, and YF performed the experiments and analyzed the data. All authors contributed to the article and approved the submitted version.

## References

[B1] Abdul Rahman N. S. N. Abdul Hamid N. W. Nadarajah K. (2021). Effects of abiotic stress on soil microbiome. Int. J. Mol. Sci. 22, 9036. 10.3390/ijms22169036 34445742 PMC8396473

[B2] Avrahami S. Bohannan B. J. (2007). Response of nitrosospira sp. strain AF-Like ammonia oxidizers to changes in temperature, soil moisture content, and fertilizer concentration. Appl. Environ. Microbiol. 73, 1166–1173. 10.1128/AEM.01803-06 17158615 PMC1828661

[B3] Barns S. M. Cain E. C. Sommerville L. Kuske C. R. (2007). Acidobacteria phylum sequences in uranium-contaminated subsurface sediments greatly expand the known diversity within the phylum. Appl. Environ. Microbiol. 73, 3113–3116. 10.1128/AEM.02012-06 17337544 PMC1892891

[B4] Berman-Frank I. Lundgren P. Falkowski P. (2003). Nitrogen fixation and photosynthetic oxygen evolution in *Cyanobacteria*. Res. Microbiol. 154, 157–164 10.1016/S0923-2508(03)00029-9 12706503

[B5] Blanchette M. L. Lund M. A. (2021). Aquatic ecosystems of the anthropocene: limnology and microbial ecology of mine pit lakes. Microorganisms 9, 1207. 10.3390/microorganisms9061207 34204924 PMC8228816

[B6] Bothe H. Schmitz O. Yates M. G. Newton W. E. (2010). Nitrogen fixation and hydrogen metabolism in *Cyanobacteria*. Microbiol. Mol. Biol. Rev. 74, 529–551. 10.1128/MMBR.00033-10 21119016 PMC3008169

[B7] Cai F. Luo P. Yang J. Irfan M. Zhang S. An N.. (2021). Effect of long-term fertilization on ammonia-oxidizing microorganisms and nitrification in brown soil of northeast China. Front. Microbiol. 11, 622454. 10.3389/fmicb.2020.622454 33613469 PMC7890093

[B8] Cai L. Guo Z. Zhang J. Gai Z. Liu J. Meng Q. (2021). No tillage and residue mulching method on bacterial community diversity regulation in a black soil region of northeastern China. PLoS ONE. 16, e0256970. 10.1371/journal.pone.0256970 34506513 PMC8432829

[B9] Cua L. S. Stein L. Y. (2011). Effects of nitrite on ammonia-oxidizing activity and gene regulation in three ammonia-oxidizing bacteria. FEMS Microbiol. Lett. 319, 169–175. 10.1111/j.1574-6968.2011.02277.x 21470297

[B10] Dang P. Yu X. Le H. Liu J. Shen Z. Zhao Z.. (2017). Effects of stand age and soil properties on soil bacterial and fungal community composition in Chinese pine plantations on the loess plateau. PLoS ONE. 12, e0186501. 10.1371/journal.pone.0186501 29049349 PMC5648195

[B11] Dawson K. S. Strapoc D. Huizinga B. Lidstrom U. Ashby M. Macalady J. L. (2012). Quantitative fluorescence in situ hybridization analysis of microbial consortia from a biogenic gas field in Alaska's cook inlet basin. Appl. Environ. Microbiol. 78, 3599–3605. 10.1128/AEM.07122-11 22427501 PMC3346356

[B12] Fan D. M. Zhao Z. M. Wang Y. Ma J. H. Wang X. C. (2022). Crop-type-driven changes in polyphenols regulate soil nutrient availability and soil microbiota. Front. Microbiol. 13, 964039. 10.3389/fmicb.2022.964039 36090073 PMC9449698

[B13] Fernandez A. L. Sheaffer C. C. Wyse D. L. Staley C. Gould T. J. Sadowsky M. J. (2016). Structure of bacterial communities in soil following cover crop and organic fertilizer incorporation. Appl. Microbiol. Biotechnol. 100, 9331–9341. 10.1007/s00253-016-7736-9 27464828

[B14] Griffith J. C. Lee W. G. Orlovich D. A. Summerfield T. C. (2017). Contrasting bacterial communities in two indigenous Chionochloa (Poaceae) grassland soils in New Zealand. PLoS ONE 12, e0179652. 10.1371/journal.pone.0179652 28658306 PMC5489180

[B15] Gui H. Fan L. Wang D. Yan P. Li X. Pang Y.. (2022). Variations in soil nutrient dynamics and bacterial communities after the conversion of forests to long-term tea monoculture Systems. Front. Microbiol. 13, 896530. 10.3389/fmicb.2022.896530 35814650 PMC9263701

[B16] Guo H. Yang L. Han X. Dai J. Pang X. Ren M.. (2019). Distribution characteristics of heavy metals in surface soils from the western area of Nansi Lake, China. Environ. Monit. Assess. 191, 262. 10.1007/s10661-019-7390-7 30949849

[B17] Huang W. Chen X. Wang K. Chen J. Zheng B. Jiang X. (2019). Comparison among the microbial communities in the lake, lake wetland, and estuary sediments of a plain river network. Microbiol. Open 8, e00644. 10.1002/mbo3.644 29888529 PMC6391271

[B18] Jin X. Rahman M. K. Ma C. Zheng X. Wu F. Zhou X. (2023). Silicon modification improves biochar's ability to mitigate cadmium toxicity in tomato by enhancing root colonization of plant-beneficial bacteria. Ecotoxicol. Environ. Safety 249, 114407. 10.1016/j.ecoenv.2022.114407 36508786

[B19] Kowalchuk G. A. Stephen J. R. (2001). Ammonia-oxidizing bacteria: a model for molecular microbial ecology. Annu, Rev, Microbiol. 55, 485–529. 10.1146/annurev.micro.55.1.485 11544365

[B20] Lin X. Green S. Tfaily M. Prakash O. Konstantinidis K. Corbett J.. (2012). Microbial community structure and activity linked to contrasting biogeochemical gradients in bog and fen environments of the glacial lake Agassiz Peatland. Appl. Environ. Microbiol. 78, 7023–7031. 10.1128/AEM.01750-12 22843538 PMC3457479

[B21] Lv X. Ma B. Yu J. Chang S. X. Xu J. Li Y.. (2016). Bacterial community structure and function shift along a successional series of tidal flats in the Yellow River Delta. Sci. Rep. 6, 36550. 10.1038/srep36550 27824160 PMC5099912

[B22] Mellado M. Vera J. (2021). Microorganisms that participate in biochemical cycles in wetlands. Can. J. Microbiol. 67, 771–788. 10.1139/cjm-2020-0336 34233131

[B23] Narendrula-Kotha R. Nkongolo K. K. (2017). Microbial response to soil liming of damaged ecosystems revealed by pyrosequencing and phospholipid fatty acid analyses. PLoS ONE. 12, e0168497. 10.1371/journal.pone.0168497 28052072 PMC5215397

[B24] Narrowe A. Angle J. Daly R. Stefanik C. Wrighton K. Miller C. S. (2017). High-resolution sequencing reveals unexplored archaeal diversity in freshwater wetland soils. Environ. Microbiol. 19, 2192–2209. 10.1111/1462-2920.13703 28217877

[B25] Nguyen H. T. Le V. Q. Hansen A. A. Nielsen J. L. Nielsen P. H. (2011). High diversityand abundance of putative polyphosphate accumulating *Tetrasphaera*-related bacteria in activated sludge systems. Fems Microbiol. Ecol. 76, 256–267. 10.1111/j.1574-6941.2011.01049.x 21231938

[B26] Pang Z. Tayyab M. Kong C. Liu Q. Liu Y. Hu C.. (2021). Continuous sugarcane planting negatively impacts soil microbial community structure, soil fertility, and sugarcane agronomic parameters. Microorganisms 9, 2008. 10.3390/microorganisms9102008 34683329 PMC8537732

[B27] Pankratov T. A. Dedysh S. N. (2010). *Granulicella* paludicola gen. nov., sp. nov., *granulicella* pectinivorans sp. nov., *granulicella* aggregans sp. nov. and granulicella rosea sp. nov., acidophilic, polymer-degrading acidobacteria from sphagnum peat bogs. Int. J. Syst. Evol. Microbiol. 60, 2951–2959. 10.1099/ijs.0.021824-0 20118293

[B28] Prasitwuttisak W. Hoshiko Y. Maeda T. Haraguchi A. Yanagawa K. (2022). Microbial community structures and methanogenic functions in wetland peat soils. Microbes Environ. 37, ME22004. 10.1264/jsme2.ME22004 35851269 PMC9530717

[B29] Quiza L. Lalonde I. Guertin C. Constant P. (2014). Land-use influences the distribution and activity of high affinity CO-oxidizing bacteria associated to type l-coxl genotype in soil. Front. Microbiol. 5, 271. 10.3389/fmicb.2014.00271 24971077 PMC4053681

[B30] Ren L. Wang P. Wang C. Chen J. Hou J. Qian J. (2017). Algal growth and utilization of phosphorus studied by combined mono-culture and co-culture experiments. Environ. Pollut. 220, 274–285. 10.1016/j.envpol.2016.09.061 27665120

[B31] Shang Y. Wu X. Wang X. Wei Q. Ma S. Sun G. (2022). Factors affecting seasonal variation of microbial community structure in Hulun Lake, China. Sci. Total Environ. 805 150294. 10.1016/j.scitotenv.2021.150294 34536882

[B32] Shang Y. Wu X. Wei Q. Dou H. Wang X. Chen J.. (2020). Total Arsenic, ph, and sulfate are the main environmental factors affecting the microbial ecology of the water and sediments in Hulun Lake, China. Front. Microbiol. 11, 548607. 10.3389/fmicb.2020.548607 33072010 PMC7541820

[B33] Shao K. Gao G. Wang Y. Tang X. Qin B. (2013). Vertical diversity of sediment bacterial communities in two different trophic states of the eutrophic Lake Taihu, China. J. Environ. Sci. (China). 25, 1186–1194. 10.1016/S1001-0742(12)60122-3 24191609

[B34] Shu F. Liu Y. Zhao Y. Wu Y. Li A. (2012). Spatio-temporal distribution of TN and TP in water and evaluation of eutrophic state of lake Nansi. Environ. Sci. 33, 3748–3752. 23323402

[B35] Sierra C. A. Trumbore S. E. Davidson E. A. Vicca S. Janssens I. (2015). Sensitivity of decomposition rates of soil organic matter with respect to simultaneous changes in temperature and moisture. J. Adv. Model. Earth Syst. 7, 335–356. 10.1002/2014MS000358

[B36] Sui X. Zhang R. Frey B. Yang L. Li M. Ni H. (2019). Land use change effects on diversity of soil bacterial, acidobacterial and fungal communities in wetlands of the Sanjiang plain, northeastern China. Sci. Rep. 9, 18535. 10.1038/s41598-019-55063-4 31811224 PMC6898332

[B37] Sui X. Zhang R. Frey B. Yang L. Liu Y. Ni H.. (2021). Soil physicochemical properties drive the variation in soil microbial communities along a forest successional series in a degraded wetland in northeastern China. Ecol. Evolut. 11, 2194–2208. 10.1002/ece3.7184 33717448 PMC7920768

[B38] Sun J. Yang L. Wei J. Quan J. Yang X. (2020). The responses of soil bacterial communities and enzyme activities to the edaphic properties of coal mining areas in central China. PLoS ONE 15, e0231198. 10.1371/journal.pone.0231198 32343698 PMC7188301

[B39] Vuko M. Cania B. Vogel C. Kublik S. Schloter M. Schulz S. (2020). Shifts in reclamation management strategies shape the role of exopolysaccharide and lipopolysaccharideproducing bacteria during soil formation. Microb. Biotechnol. 13, 584–598. 10.1111/1751-7915.13532 31920012 PMC7017822

[B40] Wang K. Ye X. Chen H. Zhao Q. Hu C. He J. (2015). Bacterial biogeography in the coastal waters of northern Zhejiang, east China sea is highly controlled by spatially structured environmental gradients. Environ. Microbiol. 17, 3898–3913. 10.1111/1462-2920.12884 25912020

[B41] Wang L. F. Xia J. Yu J. J. Yang L. Y. Zhan C. S. Qiao Y. F.. (2017). Spatial variation, pollution assessment and source identification of major nutrients in surface sediments of Nansi Lake, China. Water 9, 444. 10.3390/w9060444

[B42] Wang X. Zhang Z. Yu Z. Shen G. Cheng H. Tao S. (2020). Composition and diversity of soil microbial communities in the alpine wetland and alpine forest ecosystems on the Tibetan Plateau. Sci. Total Environ. 747, 141358. 10.1016/j.scitotenv.2020.141358 32771793

[B43] Wilms R. Köpke B. Sass H. Chang T. S. Cypionka H. Engelen B. (2006). Deep biosphere-related bacteria within the subsurface of tidal flat sediments. Environ. Microbiol. 8, 709–719. 10.1111/j.1462-2920.2005.00949.x 16584482

[B44] Wu Y. N. Xu N. Wang H. Li J. B. Zhong H. X. Dong H. Y.. (2021). Variations in the diversity of the soil microbial community and structure under various categories of degraded wetland in Sanjiang Plain, northeastern China. Land Degrad. Dev. 32, 21432–2143n. 10.1002/ldr.3872

[B45] Zhang Y. Bo G. Shen M. Shen G. Yang J. Dong S.. (2022). Differences in microbial diversity and environmental factors in ploughing-treated tobacco soil. Front. Microbiol. 13, 924137. 10.3389/fmicb.2022.924137 36171748 PMC9511222

[B46] Zhang Z. Xin L. Liang C. (2007). The analysis of hydrological characteristics and processes of ecosystem in Lake Nansi during the past 50 year. Geograph. Res. 26, 955–966. 10.11821/yj2007050012

[B47] Zheng Y. Sun Y. Zhang Z. Han C. Wang Z. Liu C.. (2023). Evaluation of the distribution and mobility of labile phosphorus in sediment profiles of Lake Nansi, the largest eutrophic freshwater lake in northern China. Chemosphere 315, 137756. 10.1016/j.chemosphere.2023.137756 36610514

