# Supplementary material for: Temperature and phosphorus: the main environmental factors affecting the seasonal variation of soil bacterial diversity in Nansi Lake Wetland
Source: Front Microbiol. 2023 Jun 30;14:1169444. doi: 10.3389/fmicb.2023.1169444 (PMC10348425; doi:10.3389/fmicb.2023.1169444)
Supplement: Supplementary file 1 [file Data_Sheet_1.zip › Table S3.docx]

Table 3. Alpha diversity indices of soil microbial community in Nansi Lake wetland

| groups | observed_species | shannon | simpson | chao1 | ACE | goods_coverage |
| --- | --- | --- | --- | --- | --- | --- |
| summer | 4635 | 10.021 | 0.99 | 5998.386 | 6112.122 | 0.961 |
| winter | 2888 | 8.848 | 0.987 | 3392.334 | 3475.462 | 0.981 |
