# Supplementary material for: Temperature and phosphorus: the main environmental factors affecting the seasonal variation of soil bacterial diversity in Nansi Lake Wetland
Source: Front Microbiol. 2023 Jun 30;14:1169444. doi: 10.3389/fmicb.2023.1169444 (PMC10348425; doi:10.3389/fmicb.2023.1169444)
Supplement: Supplementary file 1 [file Data_Sheet_1.zip › Supplementary material.docx]

**Supplementary material**

**Figure S1** Distribution coordinate map of sampling points in Nansi Lake wetland.

**Figure S2** Species accumulation boxplot, Rarefaction Curve and Rank Abundance based on OTU abundance.

**Figure S3** Results of PCoA, PCA and NMDS analysis based on species abundance.

**Figure S4** Anosim analysis showed that the differences between groups were greater than the differences within groups.

**Figure S5** MetaStat test showed the phyla with significant abundance differences between summer and winter groups.

**Figure S6** MetaStat test showed the families with significant abundance differences between summer and winter groups.

**Figure S7** Simper analysis showed bacterial groups with high contribution to seasonal differences, a. phylum; b. family; c. genus.

**Figure S8** Heat map shows the annotated functions at KEGG level 1.

**Figure S9** Heat map shows the comparison of annotated functions between summer and winter groups at KEGG level 1.

**Figure S10** Heat map shows the comparison of annotated functions between summer and winter groups at KEGG level 2.

**Figure S11** Heat map shows the comparison of annotated functions between summer and winter groups at KEGG ko.

**Figure S12** T-test shows the difference of annotated functions between summer and winter groups at KEGG ko.

**Figure S13** The result of Pearson correlation analysis between the abundance of annotated phyla (A) and genera (B) and the environmental factors.

**Figure S14** Spearman correlation analysis between alpha diversity index and environmental factors.
