# Supplementary material for: Temperature and phosphorus: the main environmental factors affecting the seasonal variation of soil bacterial diversity in Nansi Lake Wetland
Source: Front Microbiol. 2023 Jun 30;14:1169444. doi: 10.3389/fmicb.2023.1169444 (PMC10348425; doi:10.3389/fmicb.2023.1169444)
Supplement: Supplementary file 1 [file Data_Sheet_1.zip › Table 2.docx]

Table 2 Statistical analysis of soil physical and chemical indexes in Nansi Lake Wetland. The differences between seasons were analyzed by t-test. *P* < 0.05 indicates significant differences.

|  | **Groups** | **temp** | **PH** | **Moisture content** | **TP** | **AP** | **TOC** | **TN** |
| --- | --- | --- | --- | --- | --- | --- | --- | --- |
| **Mean** | group W | 8.13±1.40 | 8.28±0.57 | 0.39±0.16 | 112.37±12.79 | 23.17±3.22 | 27.40±25.29 | 1164.32±1087.62 |
|  | group S | 26.46±1.85 | 8.29±0.21 | 0.35±0.14 | 178.94±107.36 | 12.90±5.62 | 13.25±12.56 | 850.16±716.5 |
| ***p* value** | | 0.000 | 0.483 | 0.272 | 0.022 | 0.000 | 0.048 | 0.206 |
