# Supplementary material for: Temperature and phosphorus: the main environmental factors affecting the seasonal variation of soil bacterial diversity in Nansi Lake Wetland
Source: Front Microbiol. 2023 Jun 30;14:1169444. doi: 10.3389/fmicb.2023.1169444 (PMC10348425; doi:10.3389/fmicb.2023.1169444)
Supplement: Supplementary file 1 [file Data_Sheet_1.zip › Table S1.docx]

Table 1. Sampling plots and groups of soil microbial and environmental factors studies in Nansi Lake wetland

| groups | plots | subgroups | sample names | longitude and latitude |
| --- | --- | --- | --- | --- |
| group S  (summer) | plot X | SX | SX1, SX3, SX5 | 34°44′33″N, 117°44′23″E |
|  | plot S | SS | SS1, SS3, SS5 | 35°14′53″N, 116°40′35″E |
|  | plot D | SD | SD1, SD3, SD5 | 35°0′8″N, 116°44′21″E |
|  | plot T | ST | ST1, ST3, ST5 | 35°19′52″N, 116°44′21″E |
| group W  (winter) | plot X | WX | WX1, WX3, WX5 | 34°44′33″N, 117°44′23″E |
|  | plot S | WS | WS1, WS3, WS5 | 35°14′53″N, 116°40′35″E |
|  | plot D | WD | WD1, WD3, WD5 | 35°0′8″N, 116°44′21″E |
|  | plot T | WT | WT1, WT3, WT5 | 35°19′52″N, 116°44′21″E |
