# Supplementary figures and images for: Temperature and phosphorus: the main environmental factors affecting the seasonal variation of soil bacterial diversity in Nansi Lake Wetland
Source: Front Microbiol. 2023 Jun 30;14:1169444. doi: 10.3389/fmicb.2023.1169444 (PMC10348425; doi:10.3389/fmicb.2023.1169444)

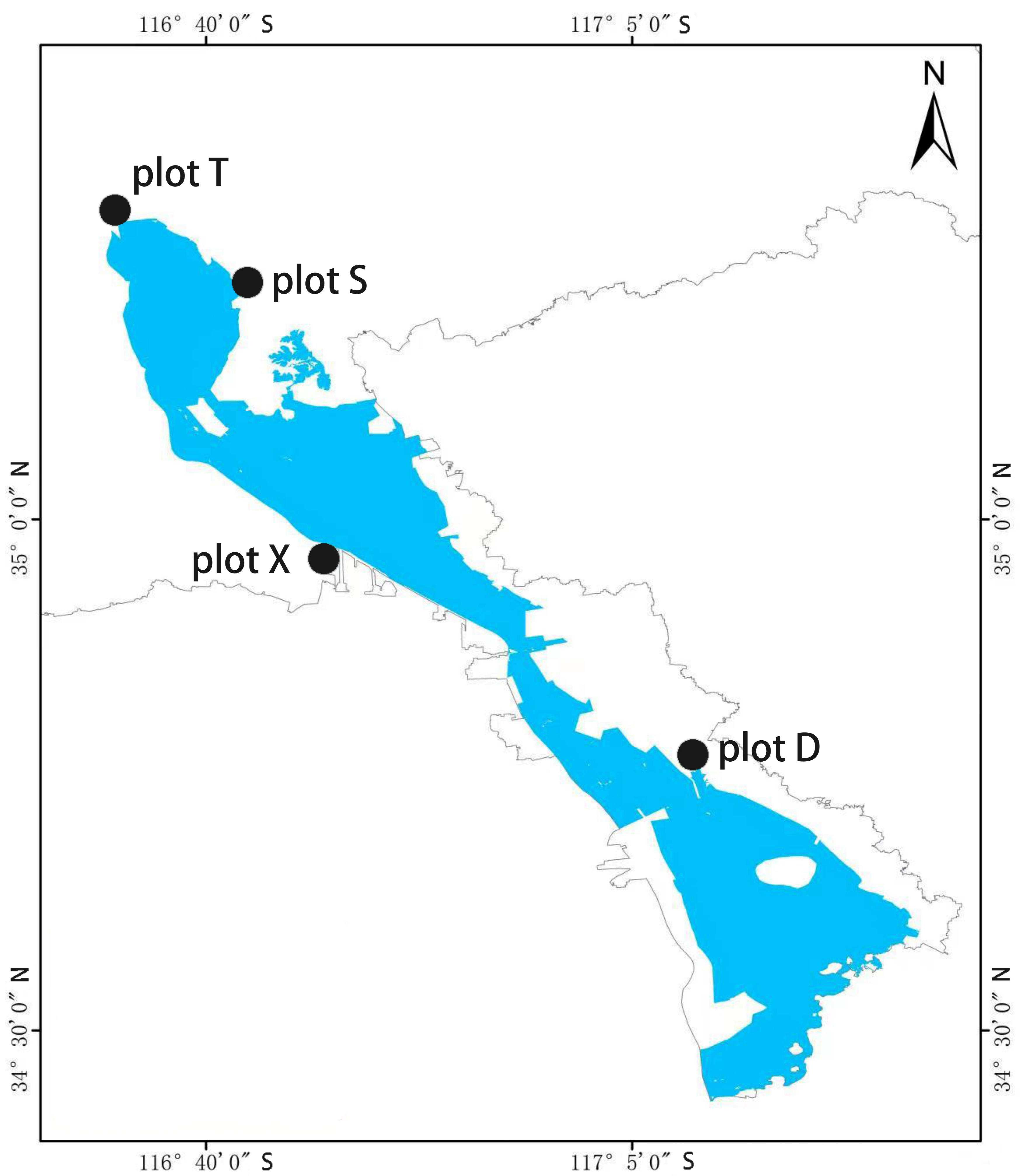

Supplement: Supplementary file 1 [file Data_Sheet_1.zip › Figure S1.pdf]

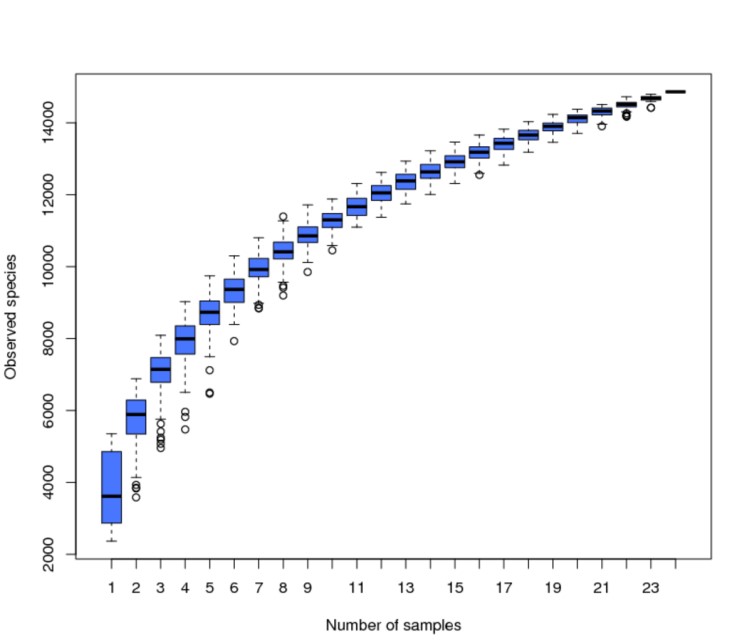

Supplement: Supplementary file 1 [file Data_Sheet_1.zip › figure S2a.jpg]

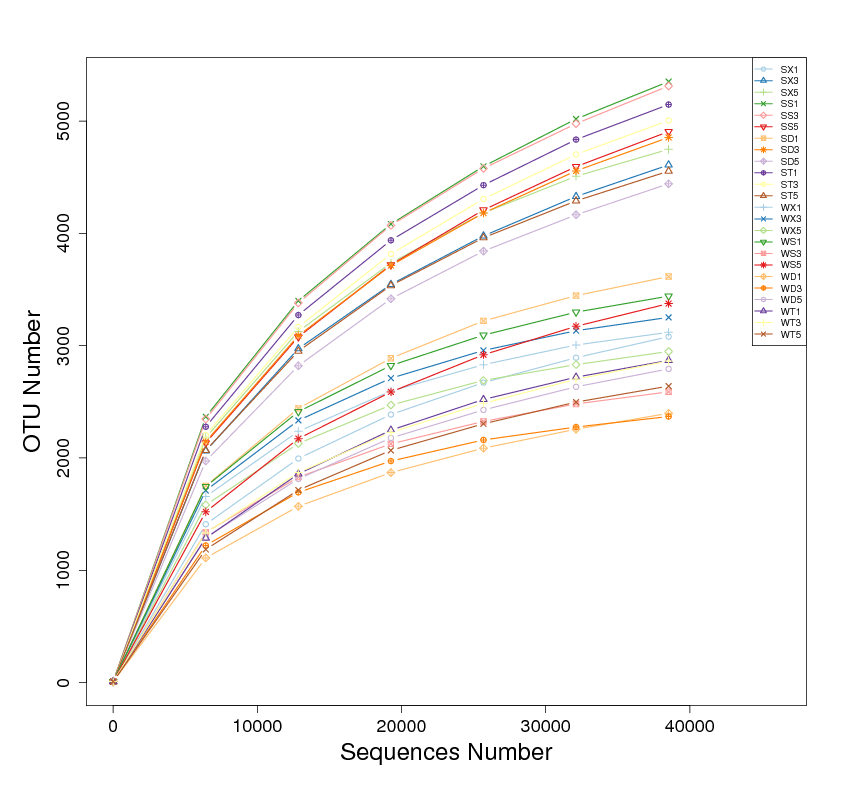

Supplement: Supplementary file 1 [file Data_Sheet_1.zip › figure S2b.png]

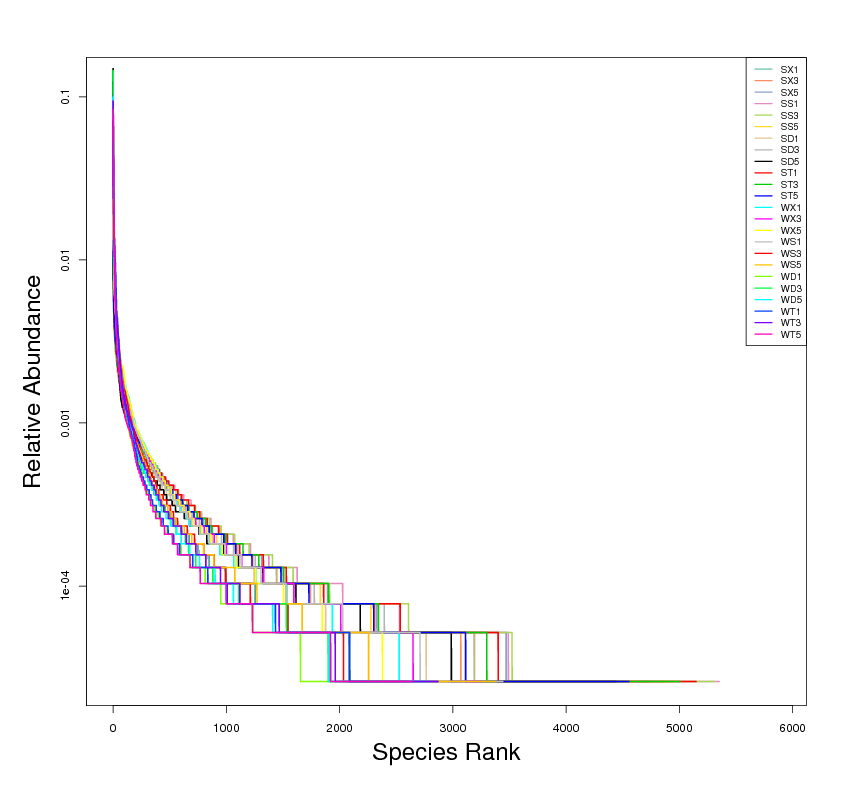

Supplement: Supplementary file 1 [file Data_Sheet_1.zip › figure S2c.png]

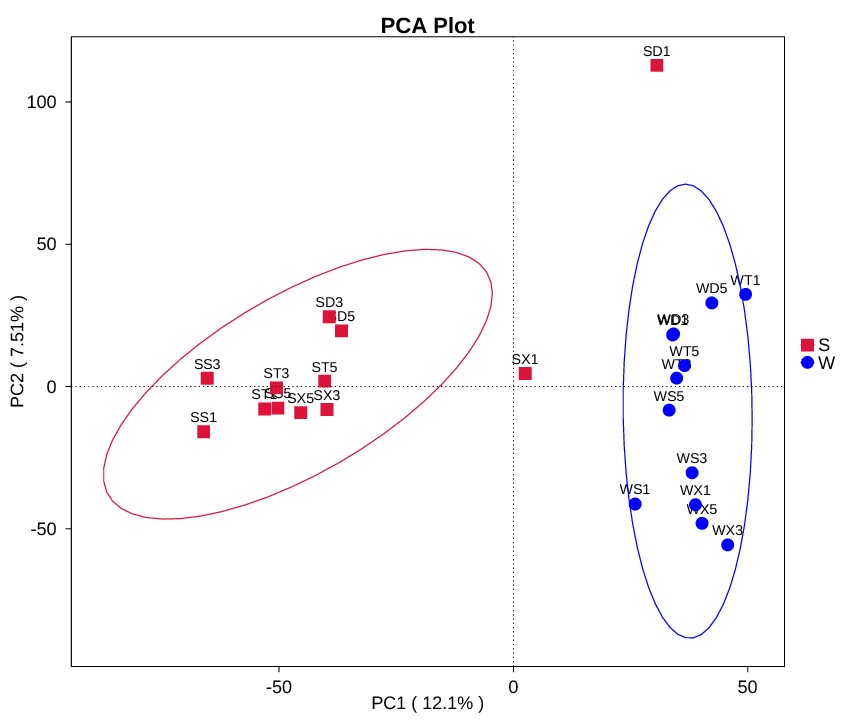

Supplement: Supplementary file 1 [file Data_Sheet_1.zip › figure S3a.jpg]

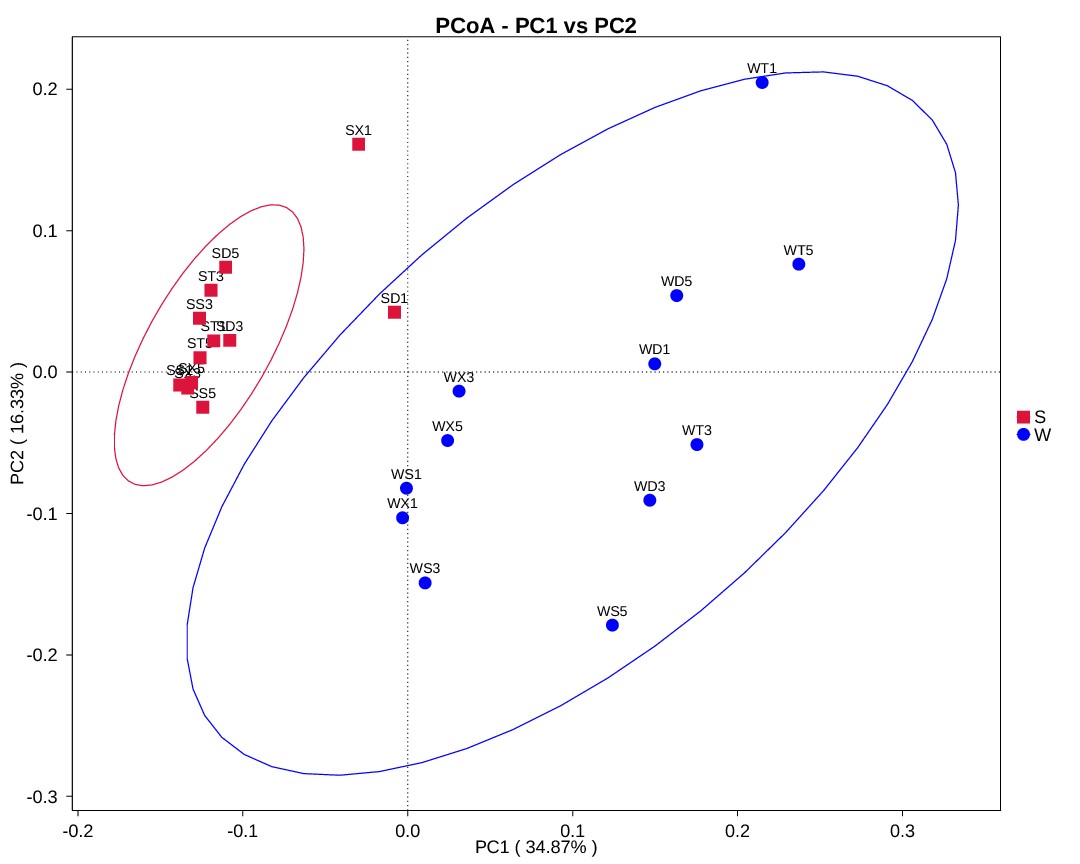

Supplement: Supplementary file 1 [file Data_Sheet_1.zip › figure S3b.jpg]

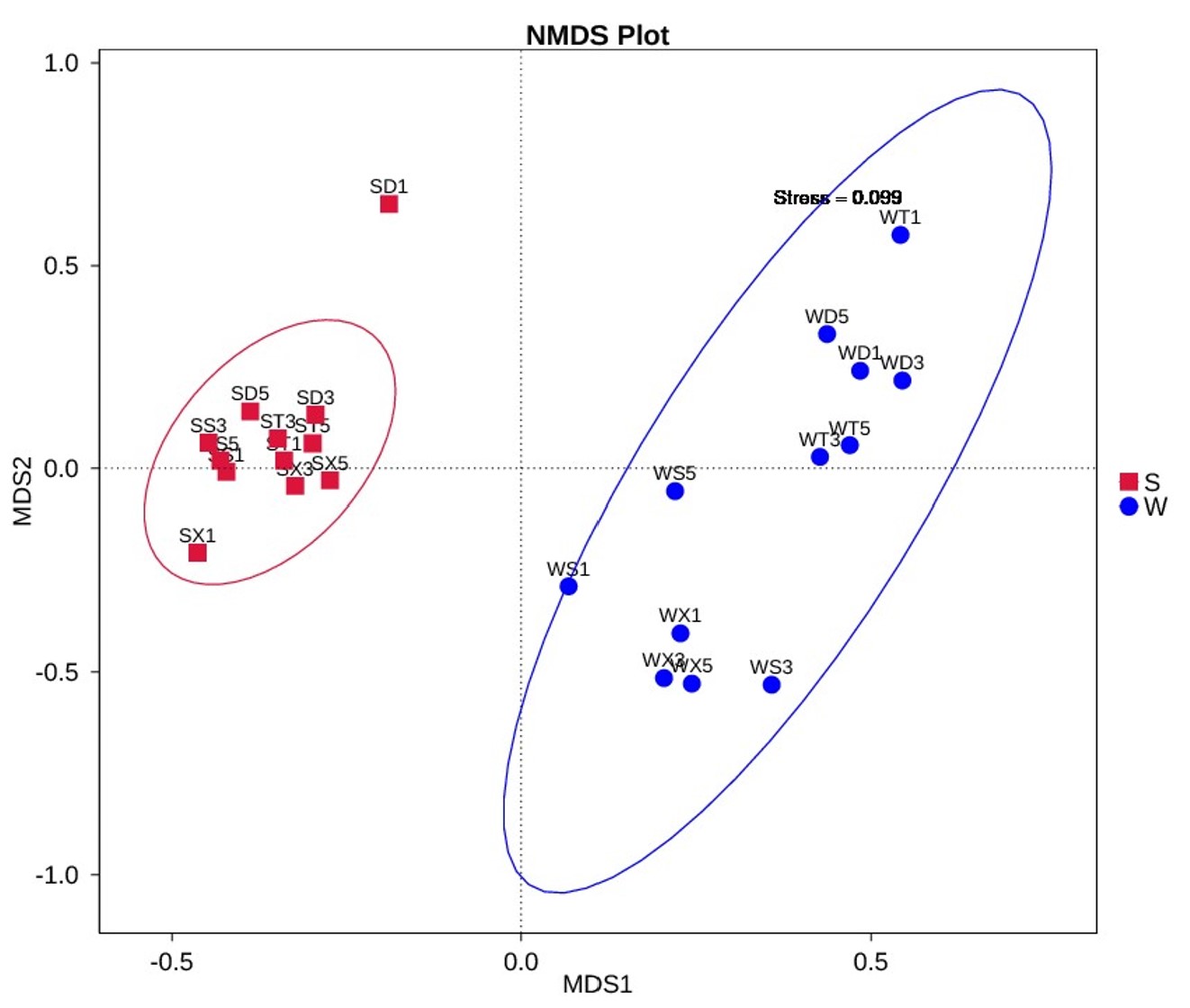

Supplement: Supplementary file 1 [file Data_Sheet_1.zip › Figure S3c.JPEG]

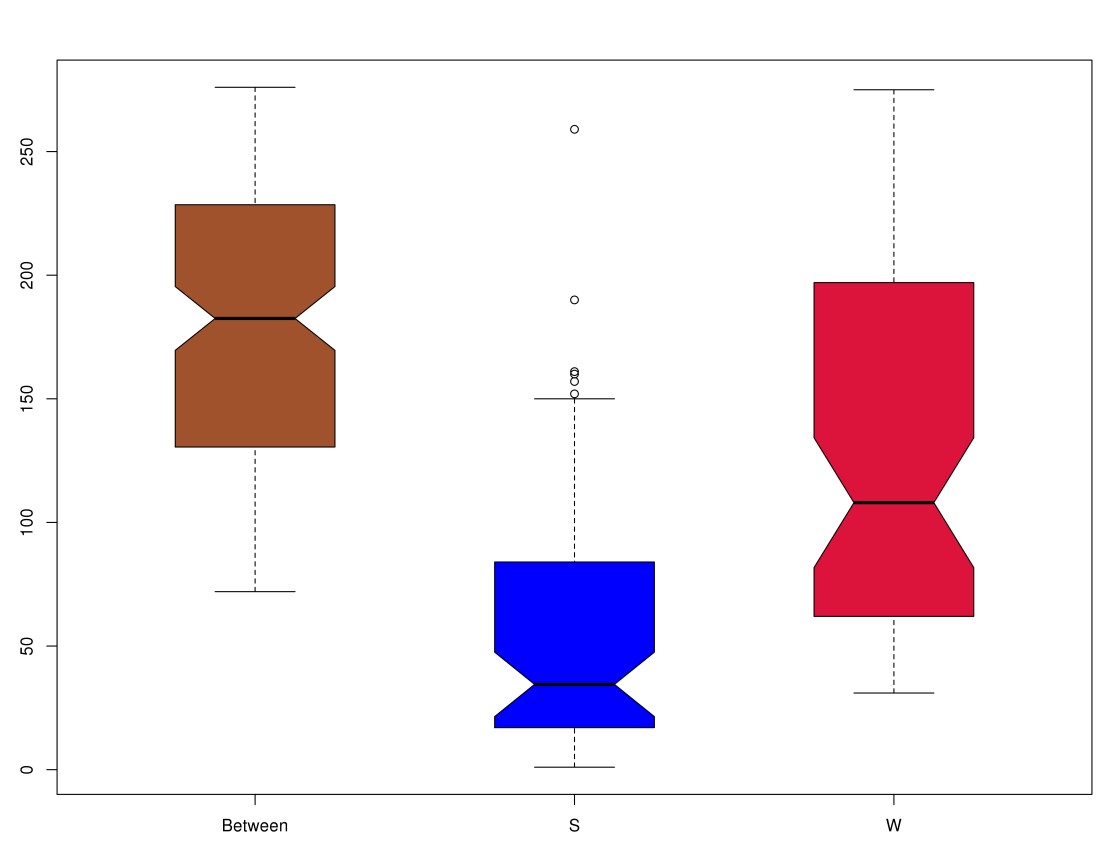

Supplement: Supplementary file 1 [file Data_Sheet_1.zip › figure S4.jpg]

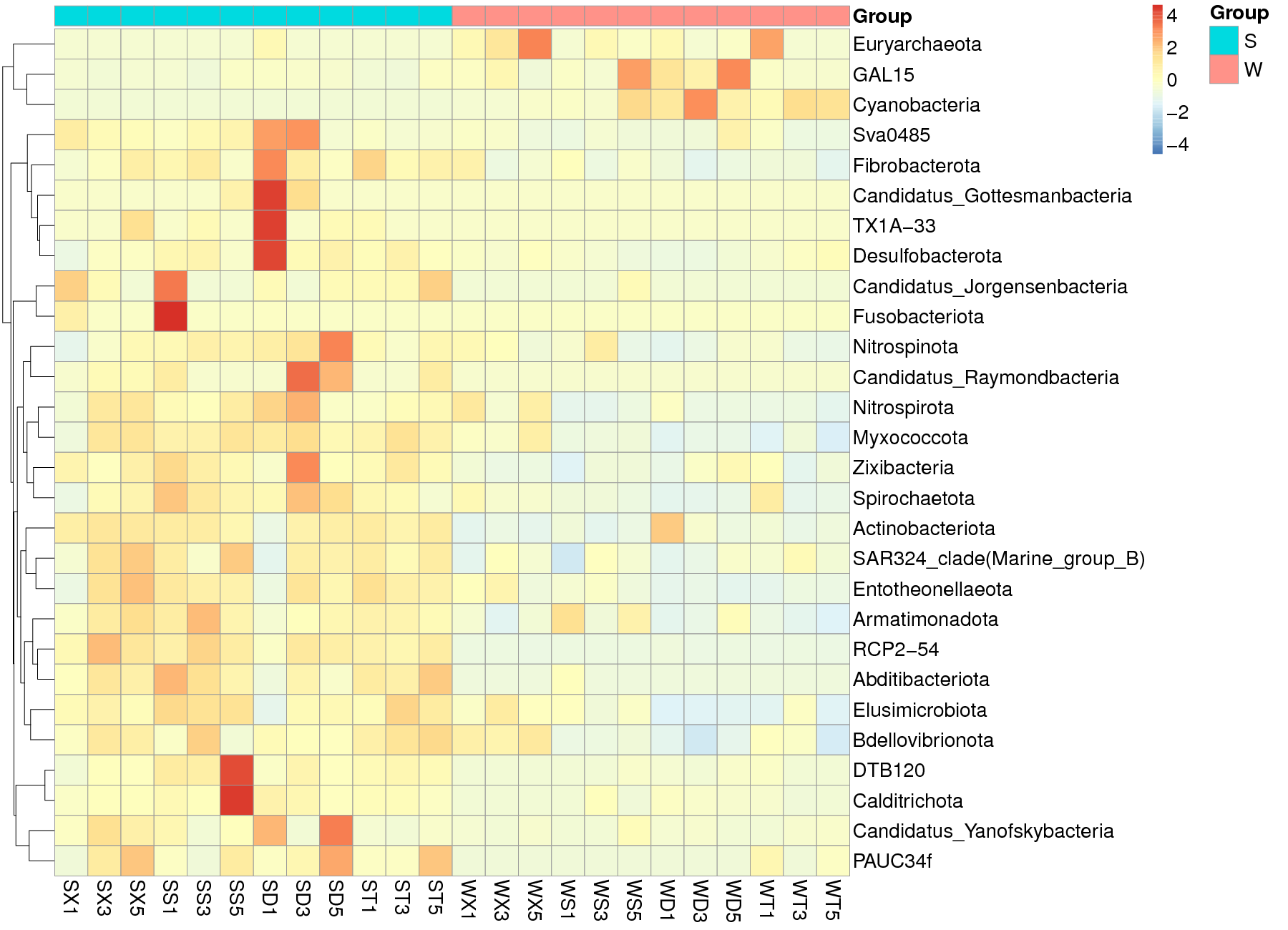

Supplement: Supplementary file 1 [file Data_Sheet_1.zip › figure S5.png]

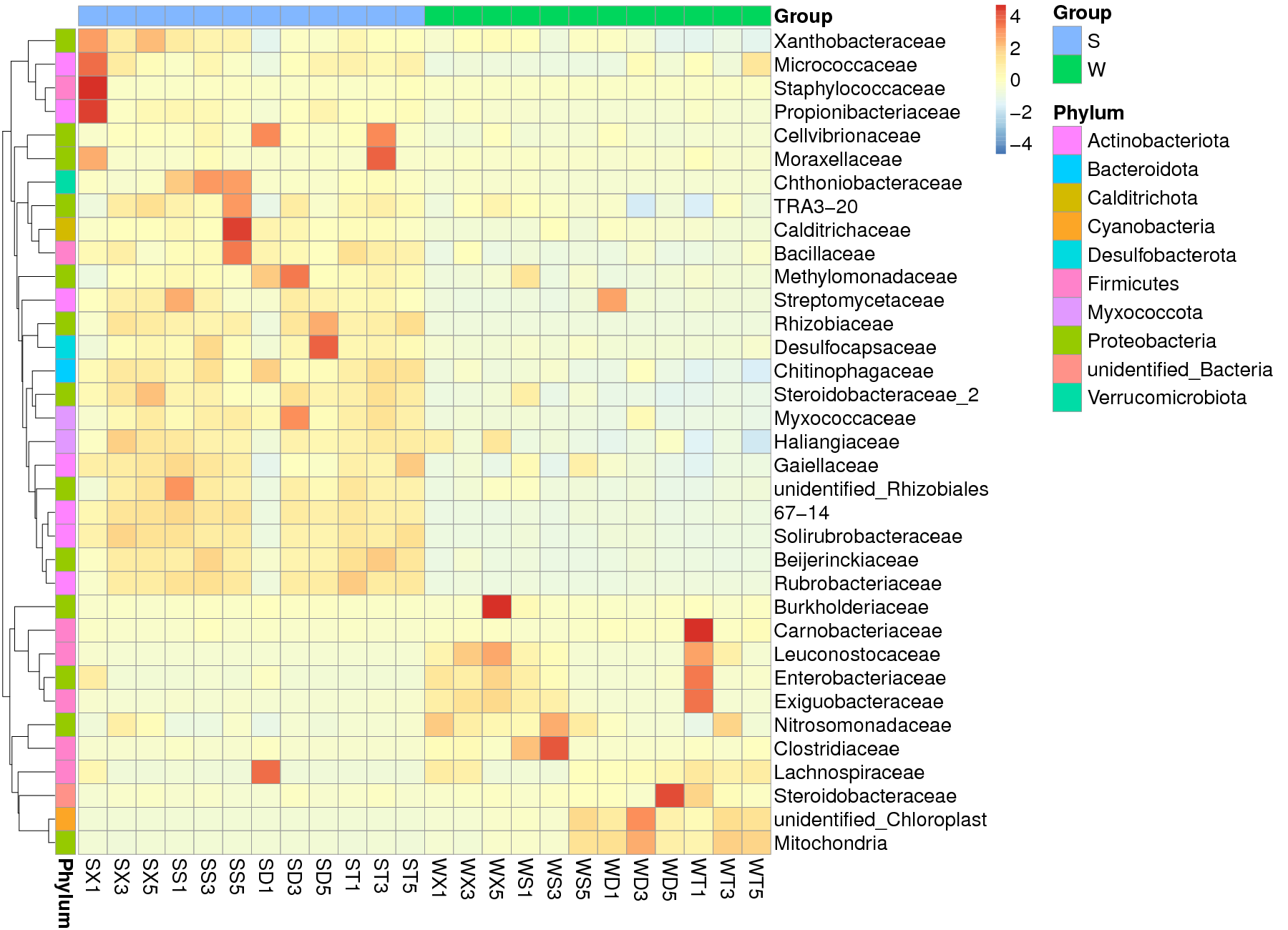

Supplement: Supplementary file 1 [file Data_Sheet_1.zip › figure S6.png]

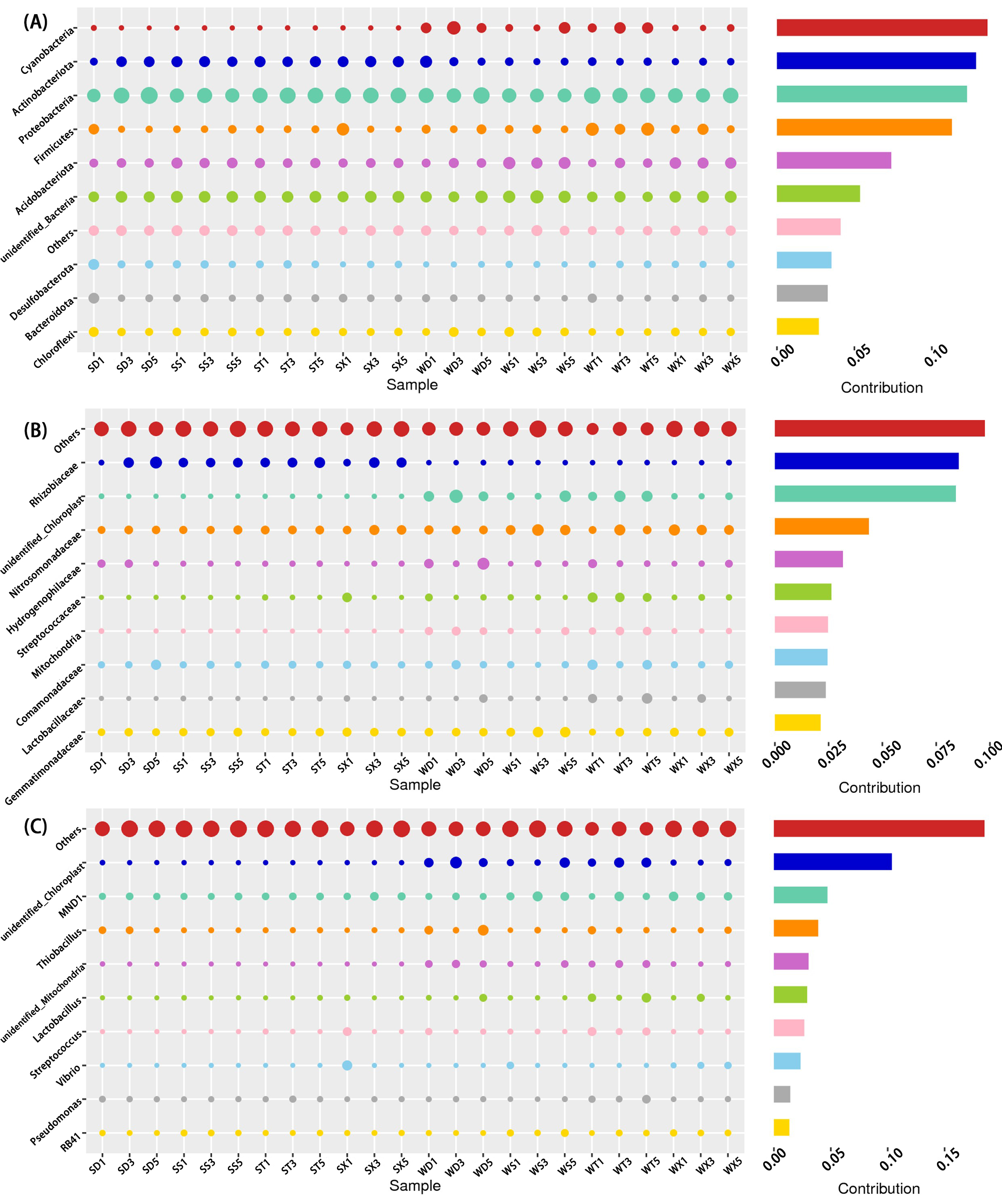

Supplement: Supplementary file 1 [file Data_Sheet_1.zip › figure S7.jpg]

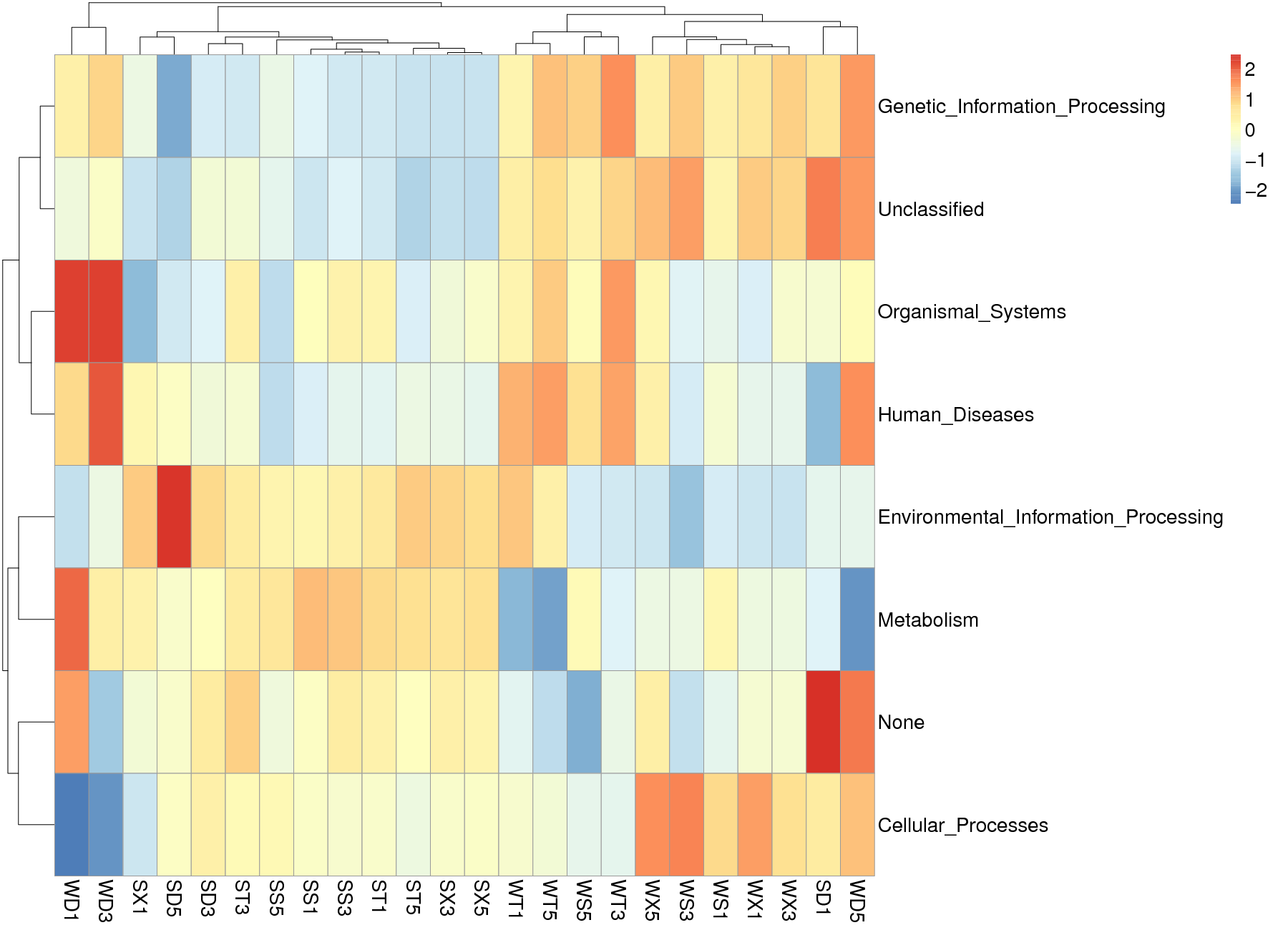

Supplement: Supplementary file 1 [file Data_Sheet_1.zip › figure S8.png]

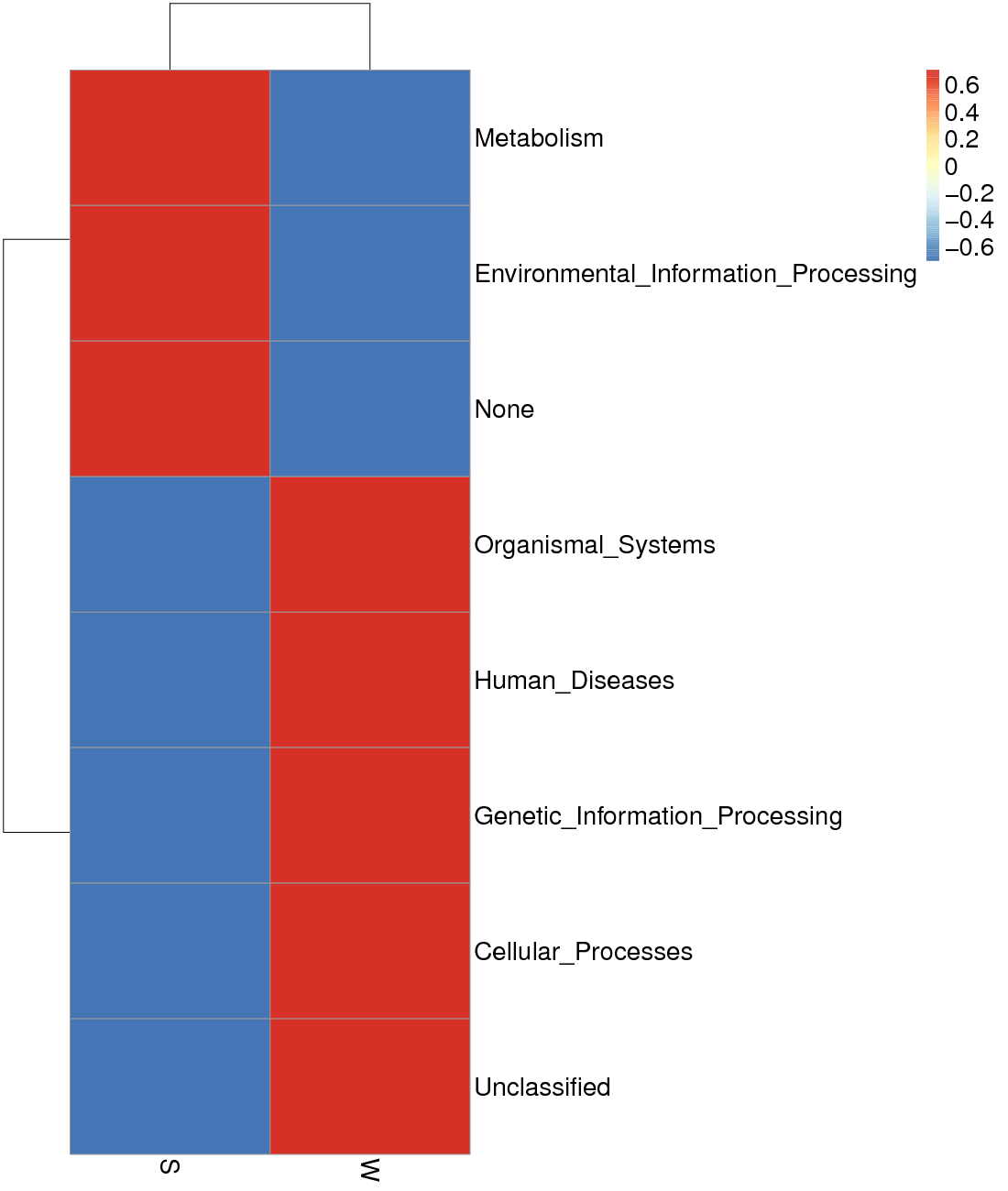

Supplement: Supplementary file 1 [file Data_Sheet_1.zip › figure S9.png]

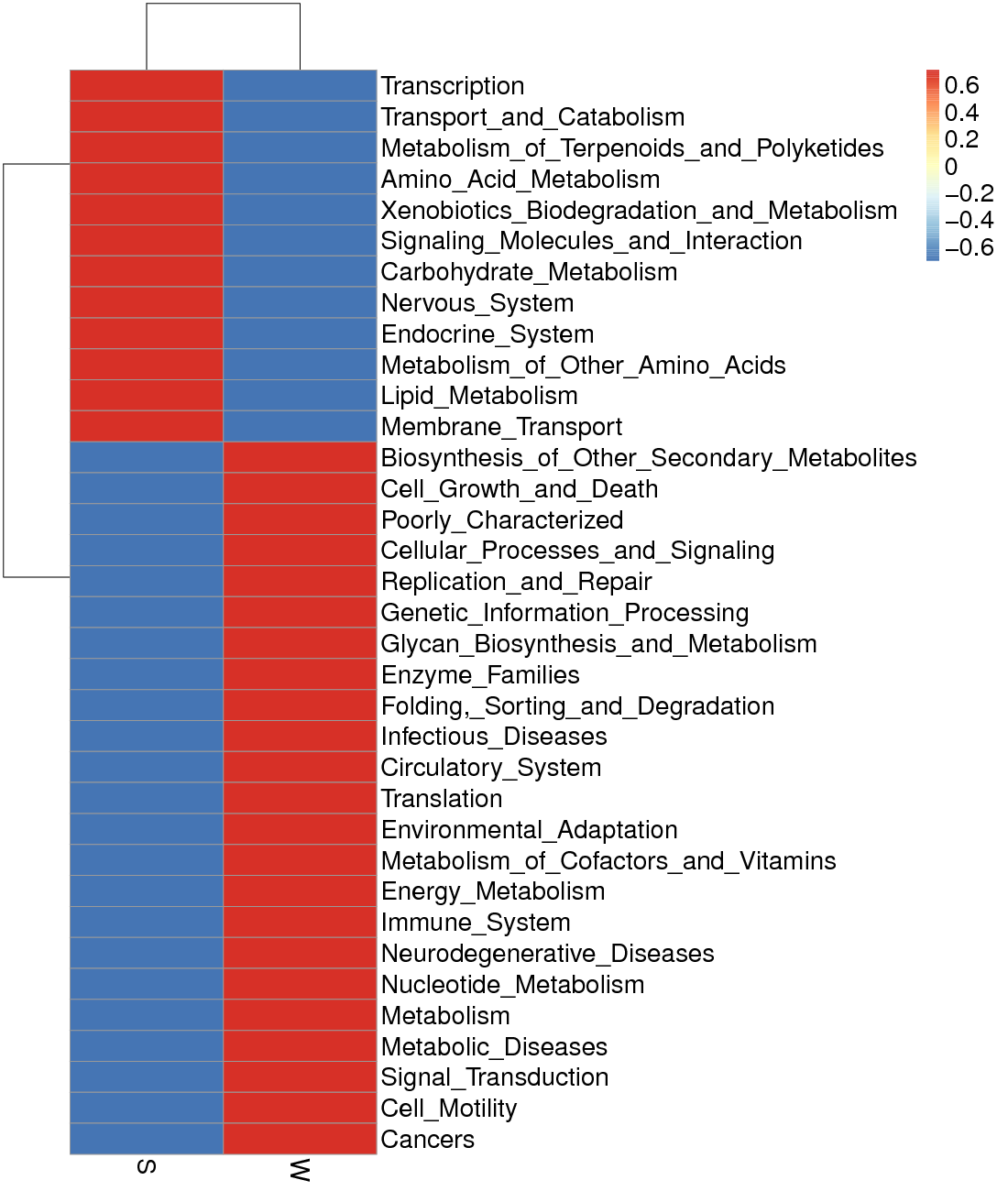

Supplement: Supplementary file 1 [file Data_Sheet_1.zip › figure S10.png]

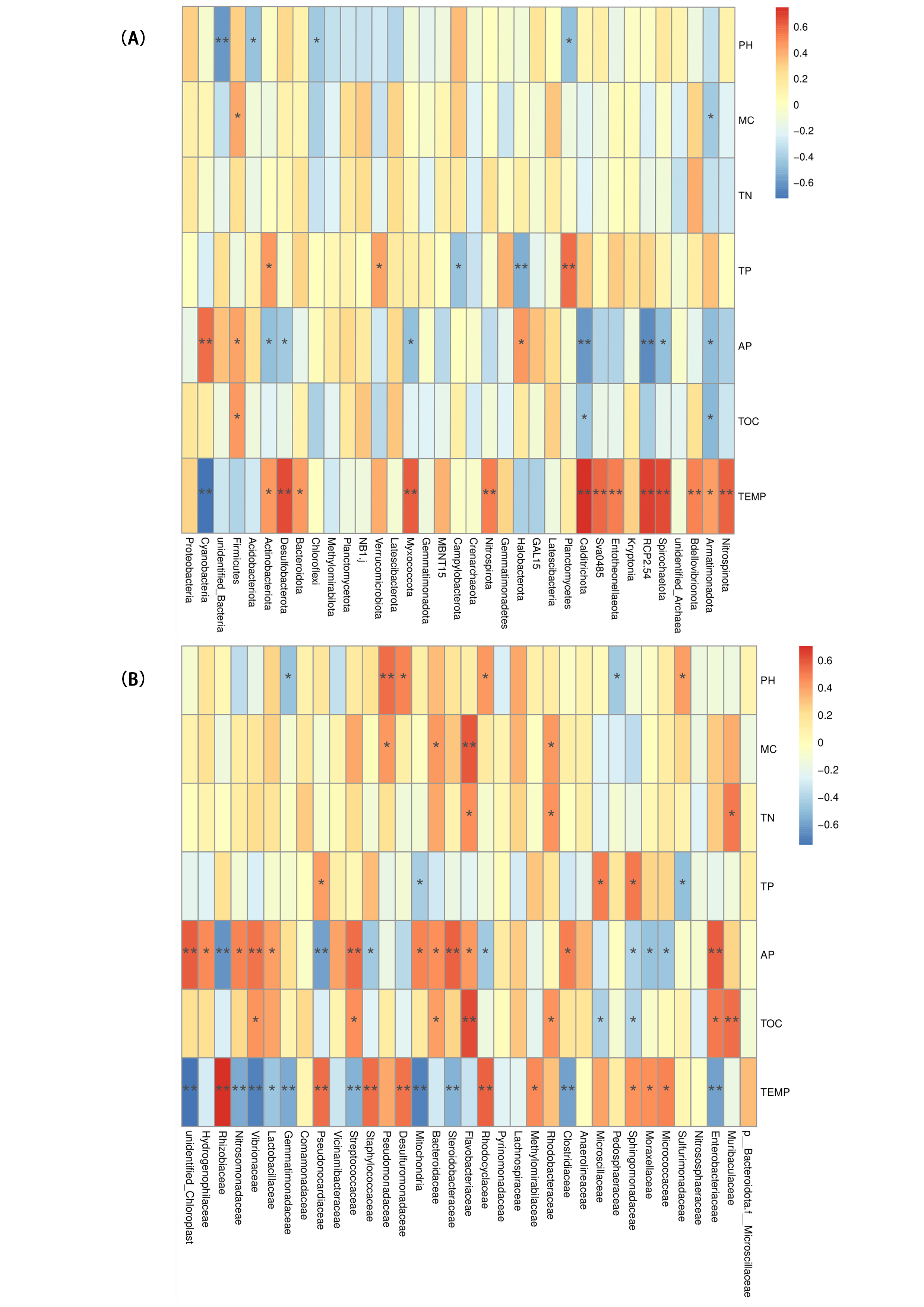

Supplement: Supplementary file 1 [file Data_Sheet_1.zip › figure S11.jpg]

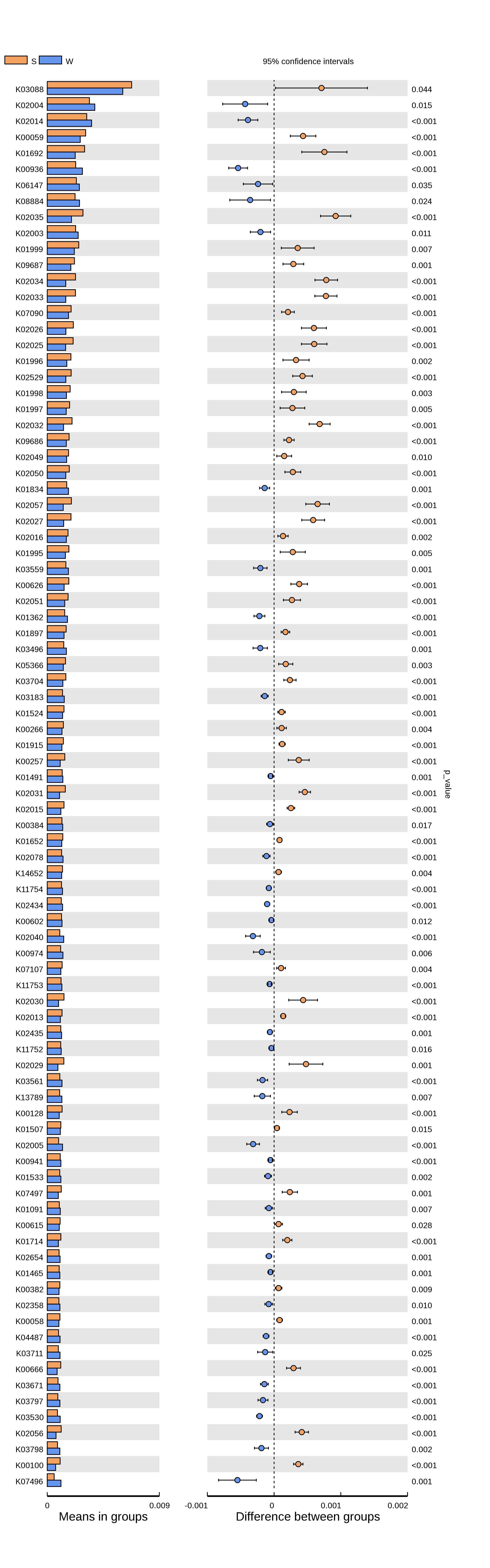

Supplement: Supplementary file 1 [file Data_Sheet_1.zip › Figure S12.png]

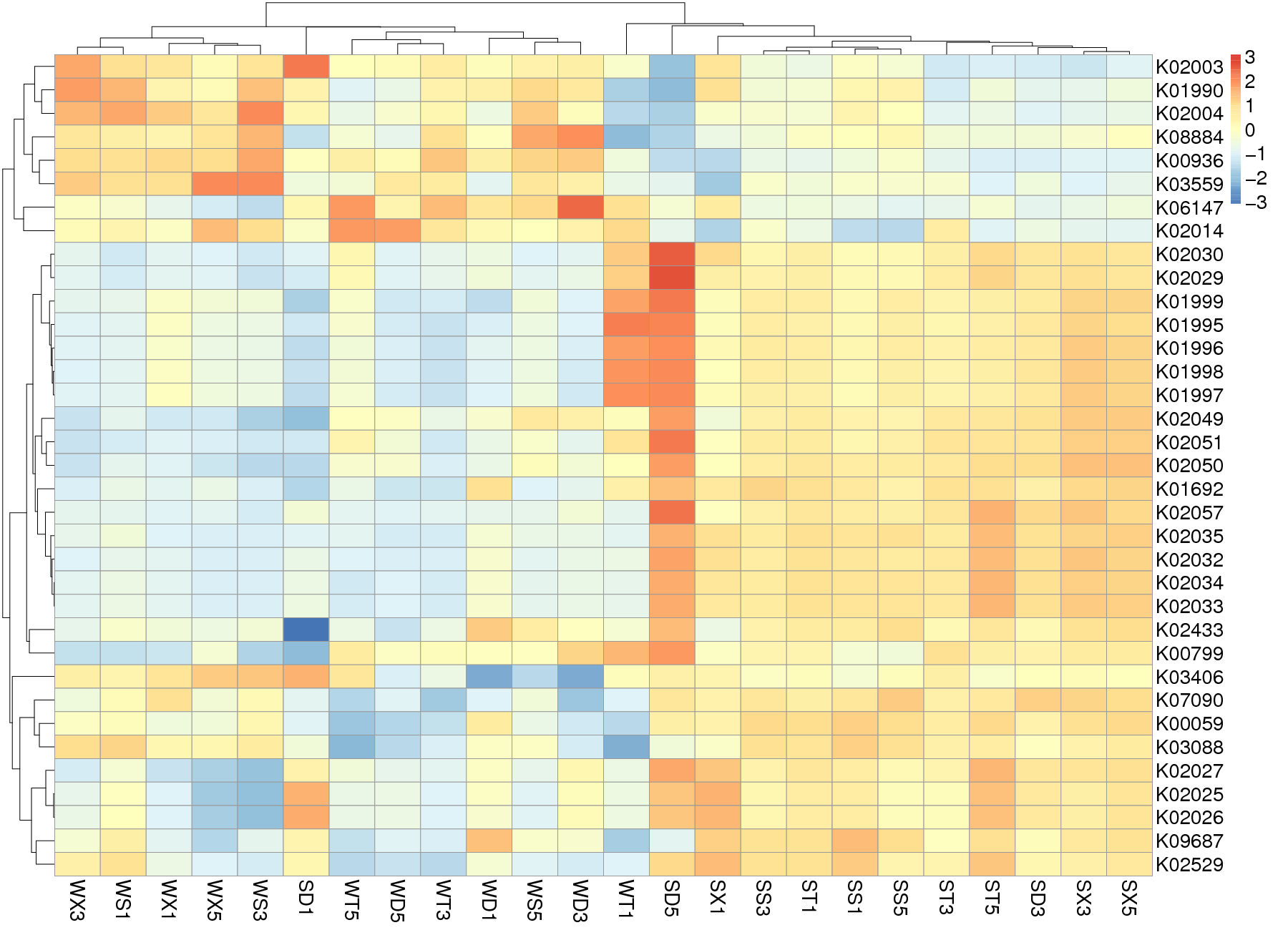

Supplement: Supplementary file 1 [file Data_Sheet_1.zip › Figure S13.png]

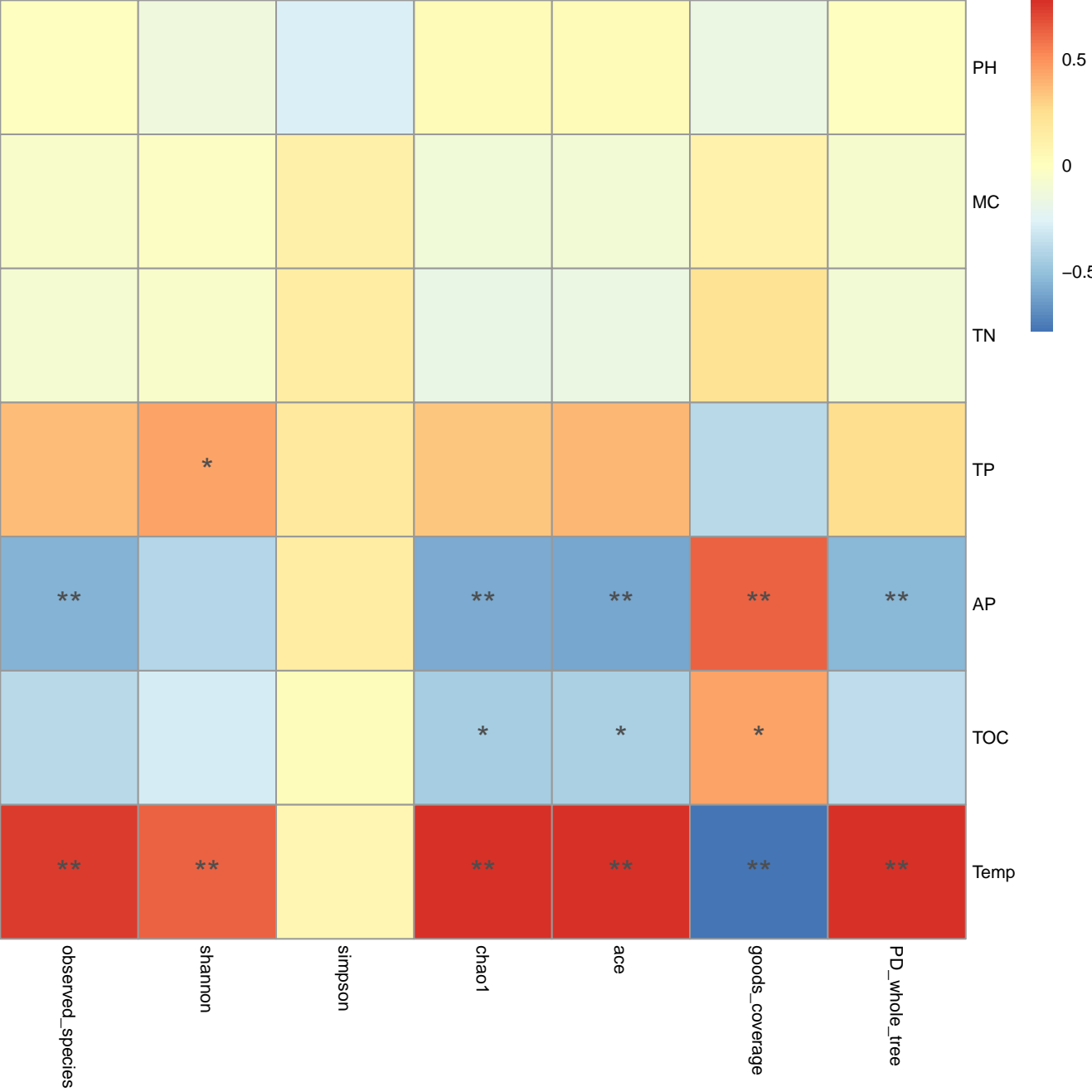

Supplement: Supplementary file 1 [file Data_Sheet_1.zip › figure S14.pdf]
